# Supplementary material for: Evaluating the impact of global fund withdrawal on needle and syringe provision, cost and use among people who inject drugs in Tijuana, Mexico: a costing analysis
Source: BMJ Open. 2019 Jan 29;9(1):e026298. doi: 10.1136/bmjopen-2018-026298 (PMC6352756; doi:10.1136/bmjopen-2018-026298)
Supplement: Supplementary file 1 [file bmjopen-2018-026298supp001.pdf]

SUPPLEMENTAL APPENDIX

Additional details on the El Cuete cohort

Epidemiological data from the El Cuete IV prospective cohort study were used to inform our findings on needle and syringe program (NSP) utilization. Study details have been described previously.[1] Briefly, eligibility criteria at baseline included injecting drugs in the past month, being at least 18 years old, and not planning to move away from Tijuana over the next 30 months. Participants were recruited by street outreach from March 2011 – May 2013 (N=734), contributing 4,301 study visits in this analysis. Participants were followed biannually, however participants were not required to have injected drugs in the previous six months at their follow-up visits. All participants provided informed consent. The study protocol was approved by the University of California San Diego Human Research Protections Program and El Colegio de la Frontera Norte (Tijuana).

Measures

Our outcome of interest was accessing a NSP (either participants themselves or from someone else who obtained syringes from a NSP) in the past six months. The independent variable of interest was the calendar period, which we classified according to its respective GF period (during and post-GF). The surveys from all visits were sorted in ascending order based on the date of the interview. Next, they were grouped into three-month periods with each period corresponding to a different GF period (during and post-GF). We note that while the actual follow-up visits were spaced approximately 6 months apart and the surveys inquired about NSP utilization in the past 6 months, the calendar periods were grouped into three-month periods to allow us to capture and estimate any seasonal variation that might have occurred within each GF period. For simplicity, in terms of when El Cuete participants may have begun noticing the impact of the GF, we classified the GF period as beginning in March 2012 and ending before June 2014 given that participants were asked about behaviors in the past 6 months. While the

dates did not align exactly with the reported GF start and end times, we assumed that it most likely took the GF sponsored activities a few months to “ramp up” and “wind down”. For example, prevention programs did not begin to be implemented in Tijuana until the third trimester of 2011.[2] In total, the data were divided into 17 evenly spaced three-month time periods, 9 of which occurred during the GF period and 8 in the post-GF period. On average, there were approximately 200-300 visits per three-month period.

Statistical analyses

We conducted segmented regression, a method used in the evaluation of intervention effects for interrupted time series data.[3] Logistic regression with fixed and random effects was used to estimate the mean log (odds of accessing a NSP) during the previous six months for each of the 17 three-month periods. Next, the mean log odds for each period estimated by the logistic regression model were used as the outcome variable in a segmented regression analysis, to predict the trend of accessing a NSP within each GF period as well as the level change from the GF period to the post-GF). Given the lack of independent error terms (as the errors of time series data are usually autocorrelated), a linear regression model was fitted to account for the autoregressive error. We tested for autocorrelation using the Breusch-Godfrey test and included first-order and second-order autoregressive terms to adjust for the effect of the positive autocorrelation.

RESULTS

The mean predicted probabilities of accessing a NSP over the 17 three-month periods are shown in Figure S1. Overall, there was a significant increasing trend in the probability of accessing the NSP during the GF period, which peaked in September 2013 (51%, 95% CI: 42% - 59%). During the GF period, the mean log odds of accessing a NSP in the increased by a factor of 0.17 (p-value < 0.001). The level change (the immediate change that occurred between the end of the last

three-months of the GF period and the end of the first three months of the post-GF period) from the GF period to the post-GF period was associated with a 0.73 reduction in the mean log odds of accessing a NSP in the past 6 months (p=0.02). During the post-GF period, the mean log odds of accessing a NSP decreased by a factor of 0.22 (p=0.002).

These trends were roughly consistent with national estimates of syringes acquired with GF support. In 2011, 534,573 syringes were distributed (92,070 financed by the GF [note: some sites may have been receiving funds prior to the NSP in Tijuana]), increasing to 1,904,961 syringes distributed (1,199,520 from the GF) in 2012. In 2013, 3,235,372 syringes were distributed with 78% provided by the GF (2,508,840). By 2014, the number of syringes distributed nationally declined to 643,320.[4]

Figure S1: Mean predicted probability of accessing a syringe exchange program in past six months among PWID in Tijuana. Error bars represent 95% confidence intervals. Shaded box represents the calendar period when the Global Fund operated in Mexico.

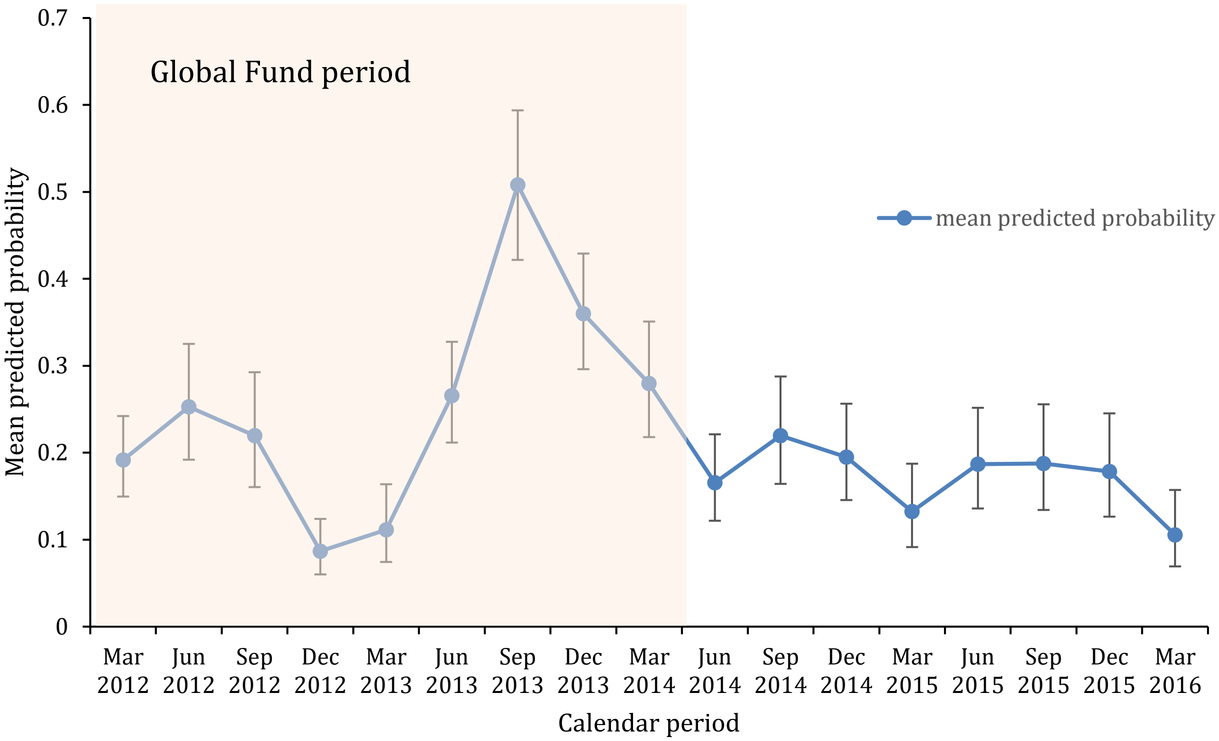

Figure S2: Number of syringes distributed in Mexico 2011 – 2014[4]

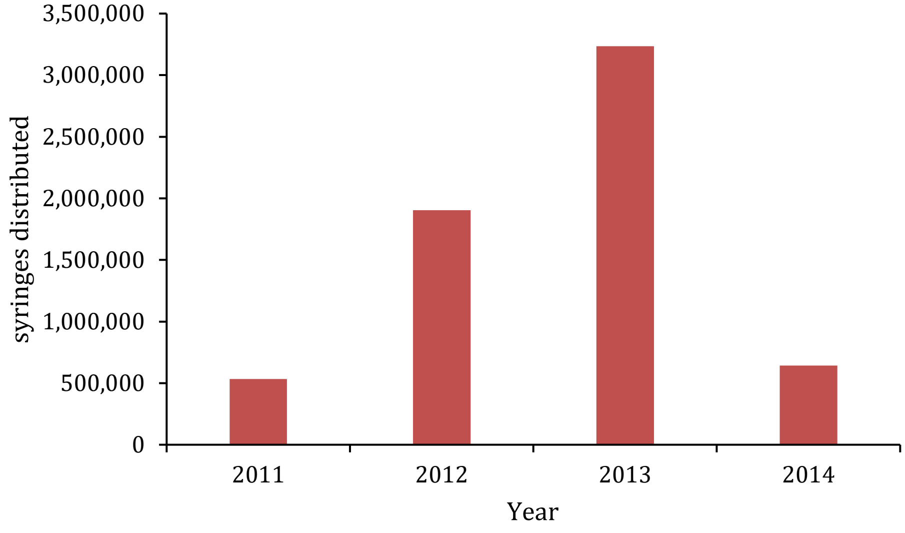

References

1. Robertson AM, Garfein RS, Wagner KD, et al. Evaluating the impact of Mexico's drug policy reforms on people who inject drugs in Tijuana, B.C., Mexico, and San Diego, CA, United States: a binational mixed methods research agenda. *Harm Reduct J.* 2014;11:4.
2. Fundación Mexicana para la Salud. "Fortalecimiento de las estrategias nacionales de prevención y reducción de daños dirigidos a HSH, HSH/TS, y UDI de ambos sexos". Plan de Implementación. 2011; http://funsalud.org.mx/vihfmfms/aprobados_fm/MX_Plan_Implentacion.pdf. Accessed 24 October, 2018.
3. Wagner AK, Soumerai SB, Zhang F, Ross-Degnan D. Segmented regression analysis of interrupted time series studies in medication use research. *Journal of clinical pharmacy and therapeutics.* 2002;27(4):299-309.
4. CENSIDA. National Report on the Progress of the HIV/AIDS Response (Informe Nacional de Avances en La Respuesta al VIH y el SIDA). 2015; http://www.censida.salud.gob.mx/descargas/ungass/GARPR_Mx2015.pdf. Accessed 25 May, 2018.
