# Supplementary material for: Evaluating the impact of global fund withdrawal on needle and syringe provision, cost and use among people who inject drugs in Tijuana, Mexico: a costing analysis
Source: BMJ Open. 2019 Jan 29;9(1):e026298. doi: 10.1136/bmjopen-2018-026298 (PMC6352756; doi:10.1136/bmjopen-2018-026298)
Supplement: Author's manuscript [file bmjopen-2018-026298.draft_revisions.pdf]

BMJ Open

BMJ Open is committed to open peer review. As part of this commitment we make the peer review history of every article we publish publicly available.

When an article is published we post the peer reviewers' comments and the authors' responses online. We also post the versions of the paper that were used during peer review. These are the versions that the peer review comments apply to.

The versions of the paper that follow are the versions that were submitted during the peer review process. They are not the versions of record or the final published versions. They should not be cited or distributed as the published version of this manuscript.

BMJ Open is an open access journal and the full, final, typeset and author-corrected version of record of the manuscript is available on our site with no access controls, subscription charges or pay-per-view fees (http://bmjopen.bmj.com).

If you have any questions on BMJ Open's open peer review process please email info.bmjopen@bmj.com

BMJ Open

Evaluating the impact of Global Fund withdrawal on syringe exchange provision, cost, and use among people who inject drugs in Tijuana, Mexico

Journal:	*BMJ Open*
Manuscript ID	bmjopen-2018-026298
Article Type:	Research
Date Submitted by the Author:	01-Sep-2018
Complete List of Authors:	Cepeda, Javier; University of California San Diego, Medicine<br>Burgos, Jose Luis; University of California San Diego, Medicine<br>Kahn, James; University of California, San Francisco, Philip R. Lee Institute for Health Policy Studies<br>Padilla, Rosario; Centro de Servicios (SER)<br>Meza, Emilio; Department of Social and Human Development<br>Segovia, Luis Alberto; Prevencasa AC<br>Gaines, Tommi; UCSD,<br>Abramovitz, Daniela; UCSD,<br>Rangel, Gudelia; El Colegio de la Frontera Norte<br>Magis, Carlos; Centro Nacional para la Prevención y el Control del VIH y el Sida (CENSIDA)<br>Vickerman, Peter; University of Bristol, School of Social and Community Medicine<br>Strathdee, Steffanie; University of California San Diego, Medicine<br>Martin, Natasha; University of California, San Diego
Keywords:	HEALTH ECONOMICS, HIV & AIDS < INFECTIOUS DISEASES, EPIDEMIOLOGY

SCHOLARONE™ Manuscripts

TITLE: Evaluating the impact of Global Fund withdrawal on syringe exchange provision, cost, and use among people who inject drugs in Tijuana, Mexico

Javier A. Cepeda[1§*], Jose Luis Burgos[1*], James G. Kahn[2], Rosario Padilla[3], Pedro Emilio Meza Martinez[4], Luis Alberto Segovia[5], Tommi Gaines[1], Daniela Abramovitz[1], Gudelia Rangel[6], Carlos Magis-Rodriguez[7], Peter Vickerman[8], Steffanie A. Strathdee[1], Natasha K. Martin[1,8]

1. Division of Infectious Diseases and Global Public Health, 9500 Gilman Drive, MC0507, University of California – San Diego, USA; jacepeda@ucsd.edu, jlburgos@ucsd.edu, togaines@ucsd.edu, dabramovitz@ucsd.edu, sstrathdee@ucsd.edu, natasha-martin@ucsd.edu

2. Institute for Health Policy Studies, School of Medicine, University of California, San Francisco, USA; jgkahn@ucsf.edu

3. Centro de Servicios (SER), Tijuana, Mexico; Gonzalez Ortega 885, Zona Centro, 22000 Tijuana, B.C., Mexico; rosario@centroser.org

4. Department of Social and Human Development, State Government of Oaxaca, Mexico; Privada Yagul 204, Universidad, San Jose la Noria, 68120 Oaxaca, Mexico; emilio.meza@gmail.com

5. Prevencasa A.C., Tijuana, Mexico; Baja California 7580, Zona Centro, 22000, Tijuana, B.C., Mexico luisalberto_segovia@yahoo.com.mx

6. El Colegio de la Frontera Norte, Carretera escénica, Km 18.5, San Antonio del Mar, 22560 Tijuana, B.C., Mexico; grangel2009@gmail.com

7. Centro Nacional para la Prevención y el Control del VIH y el Sida (CENSIDA); Av. Marina Nacional 60 , piso 8, Tacuba, Del. Miguel Hidalgo, 11410, Ciudad de México; carlos.magis@gmail.com

8. School of Social and Community Medicine, University of Bristol, Senate House, Tyndall Avenue, Bristol, BS8 1TH, UK; epxpv@bristol.ac.uk

*contributed equally

§**corresponding author:**

Javier Cepeda
Division of Infectious Diseases and Global Public Health
Department of Medicine
University of California, San Diego
9500 Gilman Drive MC 0507 La Jolla, CA, USA
Ph: 858-534-3197
jacepeda@ucsd.edu

Running head: Cost of syringe exchange in Mexico

Key words: Syringe exchange, Mexico, Global Fund, cost, harm reduction, people who inject drugs

Word count: 3280

For peer review only - http://bmjopen.bmj.com/site/about/guidelines.xhtml

For peer review only

ABSTRACT

Objective: From 2012 – 2014, the Global Fund supported syringe exchange programs in Mexico to prevent transmission of HIV among people who inject drugs. It remains unclear how Global Fund withdrawal affected the costs, quality, and coverage of syringe exchange program provision.

Design: Costing study and longitudinal cohort study.

Setting: Tijuana, Mexico

Participants: Personnel from a local syringe exchange program (N=6) and people who inject drugs (N=734) participating in a longitudinal study.

Primary outcome measures: Provision of syringe exchange program services and cost (per contact and per syringe distributed, in 2017 $USD) during Global Fund support (2012) and after withdrawal (2015/16). An additional outcome included syringe exchange program utilization from a concurrent cohort of people who inject drugs during and after Global Fund withdrawal.

Results:

During the Global Fund period, the syringe exchange program distributed 55,920 syringes to 932 contacts (60 syringes/contact) across 14 geographical locations. After Global Fund withdrew, the syringe exchange program distributed 10,700 syringes to 2,140 contacts (5 syringes/contact) across 3 geographical locations. During the Global Fund period, the cost per harm reduction contact was approximately 10-fold higher compared to after Global Fund ($44.72 vs. $3.81), however the cost per syringe distributed was nearly equal ($0.75 vs. $0.76) due to reduction in ancillary kit components. The mean log odds of accessing a syringe exchange program in the post- Global Fund period was significantly lower than during the GF period (p=0.02).

Conclusions:

Withdrawal of Global Fund support for syringe exchange program provision in Mexico was associated with a substantial drop in provision of sterile syringes, geographical coverage, and recent clean syringe utilization among people who inject drugs. Better planning is required to ensure harm reduction program sustainability is at scale after donor withdrawal.

Word count: 284

Strengths and limitations of this study

- ☐ We analyzed provision and cost data of a syringe exchange program in Tijuana, Mexico during and after Global Fund withdrawal which we used to estimate how withdrawal impact quality of the program.

- ☐ Our findings were further strengthened with the triangulation of self-reported syringe exchange program utilization data from a concurrent cohort of people who inject drugs in Tijuana.

- ☐ We were uncertain about the number of unique clients of the syringe exchange program since only the number of contacts (kits distributed) was provided.

For peer review only - http://bmjopen.bmj.com/site/about/guidelines.xhtml

BACKGROUND

The effectiveness of syringe exchange programs (SEP) in reducing transmission of HIV and hepatitis C virus (HCV) among people who inject drugs (PWID) has been well documented. Findings from a meta-analysis reported that SEPs from higher quality studies were associated with a 58% (95% CI: 0.22 – 0.81) reduction in HIV transmission.[1] Similarly, a recent Cochrane systematic review and meta-analysis found that SEPs were associated with a 23% reduction in HCV transmission (RR=0.79, 95% CI: 0.39 – 1.61), although a stronger effect was seen in Europe (RR=0.24, 95% CI: 0.09-0.62).[2] Despite the protective benefits of these services, the coverage of harm reductions services remains suboptimal, especially in low/middle income countries (LMIC)[3] where most of the HIV and HCV disease burden lies.[4]

Program evaluation, such as costing analyses, are important for budgeting and can help policymakers make evidence-based decisions with scarce resources. While LMIC would benefit the most from costing analyses of harm reduction services due to these countries having more limited resources, few economic evaluations of harm reduction services have been published in these settings. Studies conducted in Eastern Europe[5 6], Bangladesh[7], and China[8] showed that harm reduction services can be effective relative to their cost, especially within the context of nascent HIV epidemics among PWID. In Latin America, there have been no economic evaluations of harm reduction services.

Despite sharing one of the busiest land-border crossings in the world, numerous socioeconomic and health disparities separate Tijuana, Mexico from San Diego, California. Tijuana has a prominent Red-Light district and draws in drug and sex tourists primarily from the United States that has resulted in a localized HIV epidemic. It also has one of the highest concentrations of PWID in Mexico, 4-10% of whom are HIV-infected and >90% of whom are HCV antibody positive.[9 10] SEPs have been operating in Tijuana for more than 15 years, however prevention of transmission remains a challenge. The proportion accessing harm reduction

For peer review only - http://bmjopen.bmj.com/site/about/guidelines.xhtml

services (<10% in the last 6 months in 2011) is lower than the coverage recommended by the WHO[11] who defined “good coverage” as >60% of PWID contacting SEP services at least monthly in the past year.[12]

From 2012 to 2014 the Global Fund (GF) supported SEP provision in Mexico. However, due to Mexico’s rising GDP, the GF abruptly withdrew support in April 2014. It is unclear how this withdrawal affected the provision and economics of SEPs in Mexico. Our analysis had two objectives: 1) to compare SEP operations and costs between two periods, in 2012 (when SEPs were receiving funding from the GF) and in 2015 (after GF stopped funding projects in Mexico); and 2) to examine the effect of GF withdrawal on SEP access from PWID enrolled in a longitudinal cohort study in Tijuana. Findings from this analysis may inform harm reduction provision planning and donor support planning in other settings across the region, particularly those who may transition from donor funded to state-funded harm reduction provision.

METHODS

Harm reduction provision and cost data were collected from one SEP site in Tijuana, Mexico between March 2016 and February 2017. We collected cost data on SEP provision and cost during GF support (2012) and after the GF withdrew support (2015/2016). To estimate provision and costs of an efficient SEP with enhanced resources, we report outcomes during the highest volume month of GF support (May 2012). To estimate current provision and costs of SEP we report average monthly outcomes for 2015/16, and additionally report on provision during the highest volume month of 2015/16 (July 2015) for direct comparison with May 2012.

SEP Characteristics

The SEP was a fixed site located in the Zona Centro (near the “Red-Light” district, a hotspot of illicit drug use and commercial sex activity). Distribution of the number of syringes per contact was reliant upon available funding, however they were provided at no cost to the user. The SEP operated 11 months per year, and

For peer review only - http://bmjopen.bmj.com/site/about/guidelines.xhtml

provided sterile syringes, offered rapid HIV testing/counselling, and referred to hepatitis B and C testing/counselling.

Service provision data collection

During site visits, we reviewed daily logs of geographical outreach of syringe exchange activities, contents of sterile syringe kits, and operating hours. We obtained estimates on the number of contacts and number of syringes distributed per month from activity logs provided by senior staff.

Costing strategy

We costed from an economic perspective, monetizing all input resources, including staff, supplies (purchased or donated), building space, and other items. We used an ingredients-based top-down[13] micro-costing approach where overall inputs were measured at the programmatic level (i.e. we did not observe individual clients or services) separately and combined to generate total and per-client unit costs. We divided total monthly costs by two monthly outputs of interest (1) number of harm reduction contacts and (2) number of sterile syringe kits distributed. The study evaluated current implementation costs and did not consider start-up costs since the SEP has been in operation by a non-governmental organization for several years with support from the federal HIV/AIDS prevention agency in Mexico (CENSIDA).

We classified costs as recurrent (e.g. personnel and non-personnel) and capital. Personnel salaries were obtained from expenditure records, and the number of hours and percent effort dedicated to operating the SEP (including administration) were obtained from interviews with senior staff. During the GF investment period, outreach workers were paid per harm reduction kit distributed. Volunteer costs were calculated based on interviews with senior staff that reported the number of SEP-related hours and the wage a volunteer would have received if they had been employed. Recurrent non-personnel costs included supplies (including syringes and ancillary harm reduction items), building maintenance, utilities, and other services (accounting, maintenance,

For peer review only - http://bmjopen.bmj.com/site/about/guidelines.xhtml

cleaning, security, etc.). Unit prices for inputs were obtained from financial records, itemized bills/receipts, and sales catalogues. Capital costs included building space and equipment. Senior staff provided an overall monthly rent and an estimate of the proportion of building space that was attributed only to provision of SEP services, which we confirmed visually during site visits. We multiplied the rent by the proportion dedicated to SEP services to obtain the operational cost for only SEP provision. All recurrent costs associated with operating a vehicle (fuel, insurance, etc.) were obtained from expenditure records. Equipment and vehicle costs were amortized over the estimated lifespan of the item and then converted into a monthly cost. During the GF period, fuel costs for May 2012 were estimated using daily transportation logs, which were used to calculate miles driven. We used this to convert to estimated litres consumed using estimates of the vehicle's fuel economy multiplied by fuel prices (1 litre = $0.78 USD).[14]

Costing period during Global Fund

We obtained activity logs for May, 2012. Since provision of services varied during the GF period, we intentionally selected this month because it reflected a period of maximum (i.e. "ideal") provision with GF investment according to interviews with study staff which we then contrasted with current levels of provision (post-GF). These personnel costs were inflated to 2017 Mexican pesos using the consumer price index from the Instituto Nacional de Estadistica y Geografia and then converted to US dollars using the January 2017 exchange rate (20.72 Mexican pesos = 1 USD).

Costing period after Global Fund

In addition to cost data during the GF period, we also obtained data after GF withdrawal from 2015/2016 (costing period May – April). Costs were inflated to 2017 Mexican pesos using the midpoint of each cost year, and then converted to USD.

SEP access among PWID living in Tijuana

For peer review only - http://bmjopen.bmj.com/site/about/guidelines.xhtml

Since 2011, members of our research division have followed a cohort of PWID (N=734) living in Tijuana (Project "El Cuete IV") to assess trends and patterns in risk behaviours, HIV incidence, and harm reduction service utilization. Study procedures have been described elsewhere and all participants consented to study procedures.[10] To assess how GF withdrawal may have impacted clean syringe provision among PWID, we analysed data collected from March 2012 – June 2016 (roughly coinciding with the end of the costing period) to determine the proportion of El Cuete participants who reported receiving clean syringes from a SEP within the past 6 months. We applied methods from interrupted time series analysis and conducted segmented regression[15] to estimate significant temporal changes in SEP utilization during the GF period and then after the GF withdrew. We first fit a logistic regression model with fixed and random effects and a first order autoregressive correlation structure to generate the mean predicted probabilities for each quarter of the calendar year. We then fit the mean predicted probabilities into a segmented linear regression model controlling for autoregressive error to estimate the coefficients of accessing a SEP during the different GF periods. Additional details are provided in Additional file 1.

Patient involvement

Patients and the public were not involved in this research.

RESULTS

SEP provision and utilization during Global Fund Period (2012*)*

In 2012, the SEP provided harm reduction services six days per week and offered outreach services which covered a wide geographical area across Tijuana (Figure 1). Contents of the harm reduction kit included: 60 syringes (all low dead space), 240 alcohol swabs, 20 condoms, 60 3-ml vials of sterile water, 200 cotton swabs, one aluminium sheet, one 60 g tube of lubricant, and one bottle of bleach (Figure 2). In May 2012, the SEP reported 932 harm reduction contacts, resulting in 55,920 syringes distributed (Table 1). Personnel employed during this time included: a coordinator, accountant, counsellor, and nurse. Eight health education/outreach

For peer review only - http://bmjopen.bmj.com/site/about/guidelines.xhtml

workers conducted syringe exchange at various sites and 115 HIV tests were conducted. Based on the monthly activity log, we estimated that 234 km per month were travelled for outreach purposes.

Table 1: Provision of SEP services during the GF period (May, 2012), highest volume (July, 2015), and average month (2015/2016)

	GF-period May 2012	Post-GF period 2015/2016 (highest volume month)	Post-GF period 2015/2016 (average month)
**Unit of service** Harm reduction contacts per month	932	3,170	2,140
**Contents of harm reduction kit**			
Syringes	60	5	5
Sterile water	60	3	3
Alcohol swabs	240	5	5
Cotton	200	-	-
Foil	1	-	-
Bleach	1	-	-
Condoms	20	2	2
Lubricant (60 g)	1	-	-
**Syringes provided**	55,920	15,850	10,700
**Number of HIV tests conducted**	115	--†	55

†HIV testing data were not available for the highest volume month

Cost of SEP provision during Global Fund Period (2012)

The total monthly cost of SEP provision in May 2012 was $41,681 (Table 2). The cost per harm reduction contact was $44.72 while the cost per syringe distributed was $0.75. Nearly two-thirds (62%) of the total monthly costs were attributed to ancillary kit components (shown in Figure 2; $26,262) while syringes contributed 27% ($11,395) and 10% consisted of recurrent personnel, non-personnel, and capital costs. The cost of syringe distribution excluding ancillary kit components (personnel + other recurrent + capital + syringes only) during the GF period was $0.28 per syringe distributed.

For peer review only - http://bmjopen.bmj.com/site/about/guidelines.xhtml

Table 2: Capacity, optimum (May 2012) and average monthly costs of SEPs operating in Tijuana, Mexico (all costs in 2017 $USD)

	GF-period May 2012	Post-GF period 2015/2016 (average month)
**Unit of service**		
Harm reduction contacts per month	932	2,140
**Capital cost (monthly)**	**$778**	**$778**
Building/space*	$537	$537
Equipment	$241	$241
**Personnel[‡] (monthly)**	**$2,503**	**$2,407**
Coordinators	$748	$748
Accountant	$94	$94
Counselor/Head of harm reduction services	$424	$424
Clinician (Nurse/Physician)	$698	$698
Health educators/Outreach workers	$539	$443
**Non-personnel recurrent costs (monthly)**	**$38,399**	**$4,947**
Syringes	$11,395	$1,853
Supplies	$178	$178
Utilities and other services[‡]	$564	$485
Ancillary harm reduction contents	$26,262	$2,431
**Total monthly cost[§]**	**$41,681**	**$8,131**
**Cost per harm reduction contact**	$44.72	$3.80
**Cost per syringe distributed including ancillary kit contents**	$0.75	$0.76
**Cost per syringe distributed excluding ancillary kit contents**	$0.28	$0.53

[‡]only includes the amount dedicated to providing harm reduction services
[§]may not sum to total due to rounding

For peer review only

SEP provision and utilization post Global Fund (2015/16)

Operations and provision of harm reduction services differed substantially during and after the GF withdrew support in 2015/16 (Figure 1 and Table 1). As shown in Figure 1, geographic coverage of providing harm reduction services was sharply reduced after the GF withdrawal and limited mostly to the Zona Norte. Outreach personnel was reduced from 8 workers in 2012 to 4 workers in 2015/2016. Additionally, post-GF, the harm reduction kit contents were substantially reduced compared to the GF period (Table 1 and Figure 2). In 2015, the harm reduction kit contents included five syringes (three low-dead space and two high-dead space, compared to 60 low dead space syringes in 2012), three 3-ml vials of sterile water, five alcohol swabs, and three condoms. Additionally, post-GF, service provision was reduced by one day to five days per week. Despite decreased geographic coverage and reduced opening hours, the number of kits distributed per month was higher

For peer review only - http://bmjopen.bmj.com/site/about/guidelines.xhtml

in 2015 than in 2012. The SEP reported a mean of 2,140 monthly contacts in 2015/16 (3,170 contacts during highest volume month of 2015/16), compared to 932 in May 2012. However, because of the substantial decrease in syringes per kit, the total number of syringes distributed was substantially lower in 2015/16- an average of 10,700 syringes distributed per month in 2015/16. During the highest volume month in 2015/16, there were 15,850 syringes distributed compared to 55,920 in May 2012.

Cost of SEP provision post Global Fund (2015/2016)

The total monthly cost of the SEP was over 5-fold higher during 2012 compared to the average monthly cost during 2015/16 ($41,681 vs. $8,131 respectively), primarily due to the higher cost of the harm reduction kit during the GF period ($44.72 vs. $3.80 per kit, respectively). By comparison, the total monthly cost during the month of maximum provision, post-GF, was $10,193. The cost of just syringe distribution (excluding ancillary kit components) doubled from $0.28/syringe distributed in 2012 to $0.53/syringe distributed in 2015/16, mostly due to the reduction in syringes distributed and thus the higher personnel cost per syringe (Figure 3). However, after including ancillary kit components, there was no change in the cost per syringe distributed in 2015/16 ($0.76) compared to May 2012 ($0.75), because of the reduction in ancillary kit component expenditure. Similarly, there was little change in cost per syringe ($0.64) distributed when comparing to the maximum volume month in 2015/2016. Harm reduction kit (syringes + ancillary components) comprised 90% of the costs per syringe distributed during the GF period, whereas these items comprised only 51% in 2015/2016 (Figure 3).

Temporal trends in SEP access among PWID

The mean predicted probabilities of accessing a SEP over the 17 three-month periods are shown in Figure S1 (Additional file 1). Overall, there was a significant increasing trend in the probability of accessing the SEP during the GF period, which peaked in September 2013 (51%, 95% CI: 42% - 59%). During the GF period, the mean log odds of accessing a SEP increased by a factor of 0.17 (p-value < 0.001). The immediate change that occurred between the end of the last three-months of the GF period and the end of the first three months of the

For peer review only - http://bmjopen.bmj.com/site/about/guidelines.xhtml

post-GF period was associated with a 0.73 reduction in the mean log odds of accessing a SEP in the past 6 months (p=0.02). During the post-GF period, the mean log odds of accessing a SEP decreased by a factor of 0.22 (p=0.002).

DISCUSSION

Our analysis is among the first to describe the cost of providing syringe exchange services in a Latin American setting and the first to specifically compare coverage and costs of syringe exchange during versus after withdrawal of GF support. We found dramatic declines in geographical coverage and number of syringes and ancillary kit components distributed post-GF withdrawal among one SEP provider in Tijuana, with concomitant declines in reported syringe access among PWID. Excluding ancillary kit components, cost per syringe distributed doubled post-GF; total cost per syringe (including kit components) remained similar across periods as ancillary components were dramatically reduced to cut costs. We expect to use both GF ("ideal") and post-GF ("current") SEP provision costs to inform future cost-effectiveness analyses of SEPs on reducing HIV incidence in LMIC.

Despite dramatic declines in volume, quality, and geographical coverage of SEP post-GF withdrawal, it was encouraging that SEP provision in both periods covered an array of services recommended by the WHO [11]. Among these, the WHO recommends multiple delivery modalities (SEPs operated both fixed sites and mobile outreach), and referral for first aid, drug treatment, voluntary HIV testing and treatment, diagnosis and treatment of STIs. While several of these services are available to SEP clients, evidence based drug treatment programs, such as opioid substitution therapy, have not been scaled up sufficiently in Tijuana.[16] We note that the WHO recommends that harm reduction kits include needles and syringes, condoms, filters, sterile water, swabs, spoons, puncture-proof containers, acidifiers, tourniquets, bleach and other disinfectants, and education material. Many of these items were provided during the GF era, and although this provision was drastically reduced post-GF withdrawal, the kits still contained sterile water, alcohol swabs, and condoms. Future provision

For peer review only - http://bmjopen.bmj.com/site/about/guidelines.xhtml

should emphasize increasing coverage of both needles and syringes as well as ancillary kit components for maximum prevention benefit.

While the longitudinal PWID cohort provided some external validation of our findings on diminished SEP provision after GF withdrawal, some caution is warranted when attempting to triangulate these results. Participants in the study did not specify from which SEP (nor from which geographical site) they received syringes, thus we cannot conclude with any certainty that the participants received their syringes from the SEP that we analysed during and post-GF. Despite this, we still found a highly significant reduced log odds of SEP utilization in the post-GF period. Overall, this finding is consistent with national level data from CENSIDA, which reported an 80% decrease in the number of syringes distributed per PWID from 2013 (19.7 syringes per PWID) to 2014 (3.9 syringes per PWID; see Figure S2).[17]

To our knowledge, our study is the first to estimate the cost of SEP provision in Latin America. Our reported cost per syringe distributed in Tijuana after the GF withdrew support ($0.76 per syringe distributed) is less than estimates in other high-income countries, but higher than other low-middle income country (LMIC) settings. For example, an analysis in the U.S. and Canada estimated a cost of $2.28 per syringe distributed, after inflating to 2017 USD.[18] In the U.K., cost per syringe/needle distributed ranged from $0.28 - $2.17[19] with similar cost per syringe distributed in Australia ($1.94).[20] These differences may be attributed to higher personnel costs which represented 66% of the costs in U.S. and Canadian SEPs, while this comprised approximately 30-40% in Tijuana in the post GF period. Conversely, the cost per harm reduction contact in Mexico was higher than in other LMICs, including Bangladesh ($0.42 in 2017 USD)[7] , China ($0.13 in 2017 USD)[21] and Russia ($0.38 in 2017 USD).[22] Despite a similar number of syringes distributed per month personnel costs were approximately ten times lower in China compared to Mexico in the post GF period. However, we cannot determine whether this was due to greater efficiency or fewer personnel.

For peer review only - http://bmjopen.bmj.com/site/about/guidelines.xhtml

Limitations

Our study has several limitations. First, we did not collect any client cost data so we cannot provide any non-service delivery costs, such as PWID transportation to the SEP site. This distinction may be relevant when comparing 2012 to 2015 since PWID were provided more syringes per harm reduction kit in 2012 and therefore may have not needed to access the SEP as frequently (we saw a correspondingly lower number of contacts per month during this period compared to post-GF). In addition, the geographic reach post-GF was much more limited, potentially substantially increasing transport costs for PWID. We are also uncertain about the number of unique PWID who accessed the SEP during the GF period since we only obtained the number of contacts (kits distributed).

Secondly, we report on changes in SEP provision during and post-GF support from one SEP in Tijuana, however other providers (one additional SEP and pharmacies) also provide sterile syringes.[23] However, interviews with the other SEP provider indicate that the reduction in services is likely generalizable. It is legal to obtain syringes from pharmacies, however PWID often report discrimination and pharmacists' refusal to sell syringes.[24] It is possible that reductions in syringe provision seen within our SEP site due to withdrawal of GF support could have been countered by increase provision of sterile syringes from pharmacies; however, self-reported data from the longitudinal cohort of PWID do not indicate this occurred (data not shown).

CONCLUSIONS

In Tijuana, Mexico, abrupt withdrawal of GF support was associated with dramatic declines in coverage and availability of syringe exchange provision, an effective[25] and cost-effective HIV prevention intervention.[26] SEP provision during the GF period in Tijuana involved greater access to sterile syringes at a similar cost per syringe distributed while also providing abundant ancillary items that promoted safe injecting and sexual practices. The withdrawal of multilateral donors has undoubtedly left an impact on other LMIC. For example Romania, which

For peer review only - http://bmjopen.bmj.com/site/about/guidelines.xhtml

lost SEP funding from the World Bank upon joining the EU, saw a precipitous rise in new HIV infections shortly thereafter.[27] Thus, it is becoming increasingly important for donors and governments to understand the financial and programmatic implications of abrupt donor withdrawal, and to appropriately plan for transition strategies which ensure that these services are not compromised in terms of quality, coverage, and monetary value.

DECLARATIONS

Ethics approval and consent to participate

Study procedures were approved by the institutional review boards at University of California San Diego Human Research Protections Program and Universidad Autónoma de Baja California and all SEP personnel provided written informed consent. The study protocol for the El Cuete IV was approved by the University of California San Diego Human Research Protections Program and El Colegio de la Frontera Norte (Tijuana).

Consent for publication

Not applicable

Availability of data and material

Costing spreadsheets are available upon reasonable request. El Cuete IV data cannot be shared due to the need to ensure the confidentiality of participants.

Competing interests

NM has received unrestricted research grants from Gilead unrelated to this work, and honoraria from Gilead and Merck. All other authors declare no competing interests.

Funding

This study was funded by grants from the National Institute on Drug Abuse (R01DA037773, K01DA043421, T32DA023356, R01DA015612), Fogarty International Center (R25TW009343), and by the University of

For peer review only - http://bmjopen.bmj.com/site/about/guidelines.xhtml

California, San Diego, Center for AIDS Research (CFAR), an NIH-funded program (P30AI036214), which is supported by the following NIH Institutes and Centers: NIAID, NCI, NIMH, NIDA, NICHD, NHLBI, NIA, NIGMS, and NIDDK.

Authors' contributions

JC, JB, SS and NM conceived and designed the analysis and contributed to the writing of the manuscript. JC and JB conducted the analyses with input from JK, DA, TG, and PV. JB and PM collected cost data with assistance from RP, LAS, GR, and CM. All authors critically reviewed and approved the final version.

Acknowledgements

We thank the staff from the SEP and all respondents from the El Cuete IV study for participating.

For peer review only

For peer review only - http://bmjopen.bmj.com/site/about/guidelines.xhtml

REFERENCES

1. Aspinall EJ, Nambiar D, Goldberg DJ, et al. Are needle and syringe programmes associated with a reduction in HIV transmission among people who inject drugs: a systematic review and meta-analysis. *International journal of epidemiology* 2014;43(1):235-48. doi: 10.1093/ije/dyt243
2. Platt L, Minozzi S, Reed J, et al. Needle syringe programmes and opioid substitution therapy for preventing hepatitis C transmission in people who inject drugs. *The Cochrane database of systematic reviews* 2017;9:Cd012021. doi: 10.1002/14651858.CD012021.pub2 [published Online First: 2017/09/19]
3. Harm Reduction International. The Global State of Harm Reduction 2016, 2016.
4. Mathers BM, Degenhardt L, Phillips B, et al. Global epidemiology of injecting drug use and HIV among people who inject drugs: a systematic review. *The Lancet* 2008;372(9651):1733-45.
5. Kumaranayake L, Vickerman P, Walker D, et al. The cost-effectiveness of HIV preventive measures among injecting drug users in Svetlogorsk, Belarus. *Addiction* 2004;99(12):1565-76. doi: 10.1111/j.1360-0443.2004.00899.x
6. Vickerman P, Kumaranayake L, Balakireva O, et al. The cost-effectiveness of expanding harm reduction activities for injecting drug users in Odessa, Ukraine. *Sexually transmitted diseases* 2006;33(10 Suppl):S89-102. doi: 10.1097/01.olq.0000221335.80508.fa
7. Guinness L, Vickerman P, Quayyum Z, et al. The cost-effectiveness of consistent and early intervention of harm reduction for injecting drug users in Bangladesh. *Addiction* 2010;105(2):319-28. doi: 10.1111/j.1360-0443.2009.02755.x
8. Zhang L, Yap L, Xun Z, et al. Needle and syringe programs in Yunnan, China yield health and financial return. *BMC public health* 2011;11:250. doi: 10.1186/1471-2458-11-250
9. White EF, Garfein RS, Brouwer KC, et al. Prevalence of hepatitis C virus and HIV infection among injection drug users in two Mexican cities bordering the US. *Salud Publica Mexico* 2007;49(3):165-72. doi: Doi 10.1590/S0036-36342007000300001
10. Robertson AM, Garfein RS, Wagner KD, et al. Evaluating the impact of Mexico's drug policy reforms on people who inject drugs in Tijuana, B.C., Mexico, and San Diego, CA, United States: a binational mixed methods research agenda. *Harm Reduct J* 2014;11:4. doi: 10.1186/1477-7517-11-4
11. World Health Organization. Guide to starting and managing needle and syringe programmes. 2007
12. WHO and UNODC. Technical Guide for countries to set targets for universal access to HIV prevention, treatment and care for injecting drug users. 2009
13. Cunnama L, Sinanovic E, Ramma L, et al. Using Top-down and Bottom-up Costing Approaches in LMICs: The Case for Using Both to Assess the Incremental Costs of New Technologies at Scale. *Health Economics* 2016;25:53-66. doi: 10.1002/hec.3295
14. Dibble S. Gas price protest leads to shortages at Tijuana stations San Diego Union Tribune2017 [Available from: http://www.sandiegouniontribune.com/news/border-baja-california/sd-me-gas-shortage-tjuana-20170106-story.html accessed 12 April 2017.
15. Wagner AK, Soumerai SB, Zhang F, et al. Segmented regression analysis of interrupted time series studies in medication use research. *Journal of clinical pharmacy and therapeutics* 2002;27(4):299-309.
16. Werb D, Wagner K, Beletsky L, et al. Police bribery and access to methadone maintenance therapy within the context of drug policy reform in Tijuana, Mexico. *Drug Alcohol Depen* 2015;148:221-25.
17. CENSIDA. National Report on the Progress of the HIV/AIDS Response (Informe Nacional de Avances en La Respuesta al VIH y el SIDA) 2015 [Available from: http://www.censida.salud.gob.mx/descargas/ungass/GARPR_Mx2015.pdf accessed 25 May 2018.
18. Lurie P, Reingold AL. The Public Health Impact of Needle Exchange Programs in the United States and Abroad: Summary, Conclusions, and Recommendations. 1993
19. Platt L, Sweeney S, Ward Z, et al. Assessing the impact and cost-effectiveness of needle and syringe provision and opioid substitution therapy on hepatitis C transmission among people who inject drugs in the UK: an analysis of pooled data sets and economic modelling. 2017

For peer review only - http://bmjopen.bmj.com/site/about/guidelines.xhtml

20. Wilson D, Kwon A, Anderson J, et al. Return on investment 2: Evaluating the cost-effectiveness of needle and syringe programs in Australia. *Sydney: National Centre in HIV Epidemiology and Clinical Research, The University of New South Wales* 2009
21. Masaki E. CJ, Zhu Q., Chen X., Chen Y., Lu M. Cost -Effectiveness of Harm Reduction Interventions in Guangxi Zhuang Autonomous Region, China. *World Bank* 2007
22. Marseille E, Dandona L, Saba J, et al. Assessing the efficiency of HIV prevention around the world: methods of the PANCEA project. *Health services research* 2004;39(6p2):1993-2012.
23. Pollini RA, Lozada R, Gallardo M, et al. Barriers to Pharmacy-Based Syringe Purchase Among Injection Drug Users in Tijuana, Mexico: A Mixed Methods Study. *Aids Behav* 2010;14(3):679-87. doi: 10.1007/s10461-010-9674-3
24. Davidson PJ, Lozada R, Rosen PC, et al. Negotiating access: social barriers to purchasing syringes at pharmacies in Tijuana, Mexico. *Int J Drug Policy* 2012;23(4):286-94. doi: 10.1016/j.drugpo.2012.05.001
25. Hurley SF, Jolley DJ, Kaldor JM. Effectiveness of needle-exchange programmes for prevention of HIV infection. *The Lancet* 1997;349(9068):1797-800.
26. Wilson DP, Donald B, Shattock AJ, et al. The cost-effectiveness of harm reduction. *Int J Drug Policy* 2015;26 Suppl 1:S5-11. doi: 10.1016/j.drugpo.2014.11.007
27. Csete J, Kamarulzaman A, Kazatchkine M, et al. Public health and international drug policy. *Lancet* 2016;387(10026):1427-80. doi: 10.1016/S0140-6736(16)00619-X [published Online First: 2016/03/30]

For peer review only

For peer review only - http://bmjopen.bmj.com/site/about/guidelines.xhtml

FIGURES

Figure 1: Geographical coverage of syringe exchange outreach sites for SEP Site 1 (a) during GF period in May 2012 and (b) after GF withdrawal in 2015/16

Figure 2: Harm reduction kit components per sterile syringe distributed during the Global Fund Period and after withdrawal of Global Fund

Figure 3: Cost breakdown per syringe distributed at each SEP site (ancillary harm reduction components in brackets)

For peer review only

For peer review only - http://bmjopen.bmj.com/site/about/guidelines.xhtml

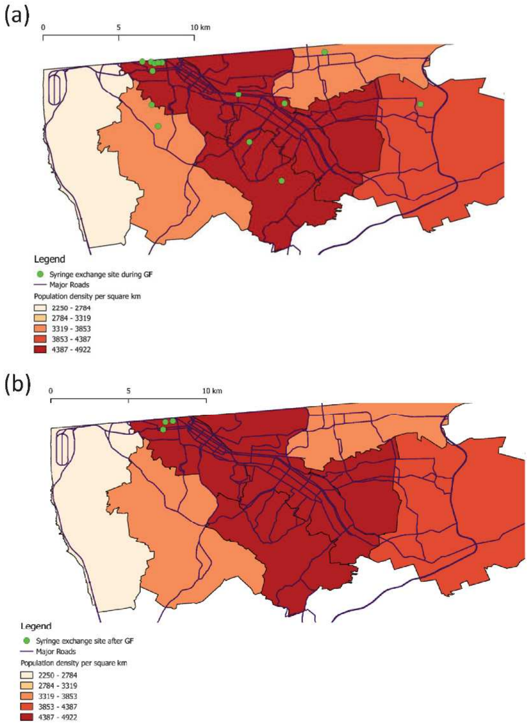

Figure 1: Geographical coverage of syringe exchange outreach sites for SEP Site 1 (a) during GF period in May 2012 and (b) after GF withdrawal in 2015/16

254x190mm (300 x 300 DPI)

For peer review only

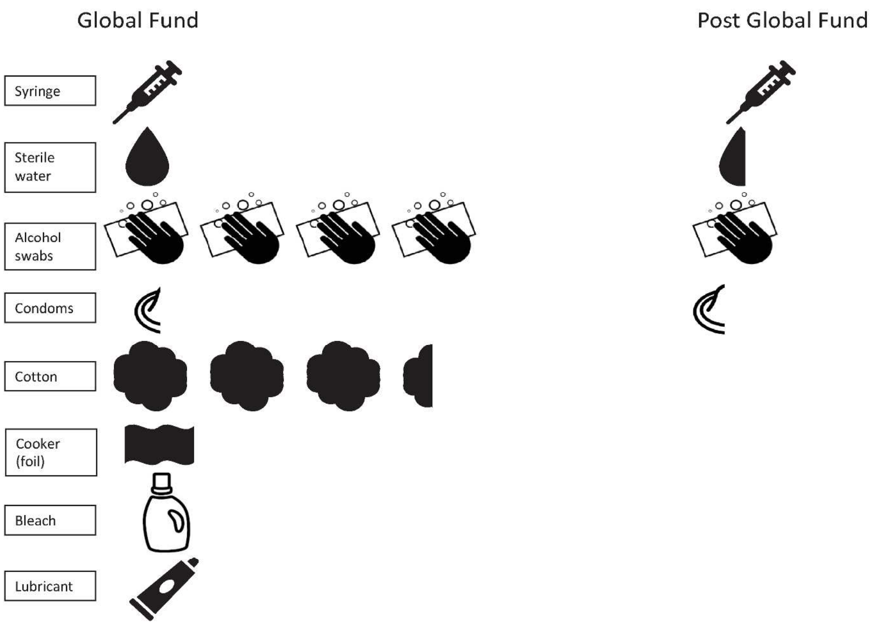

Figure 2: Harm reduction kit components per sterile syringe distributed during the Global Fund Period and after withdrawal of Global Fund

254x190mm (300 x 300 DPI)

w only

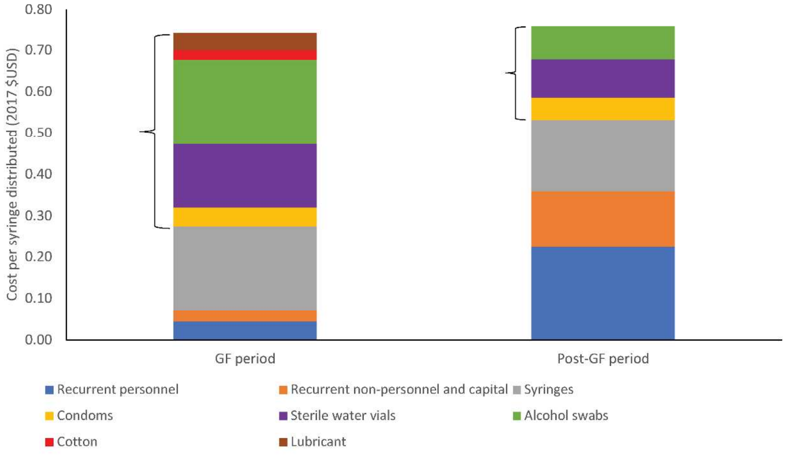

Figure 3: Cost breakdown per syringe distributed at each SEP site (ancillary harm reduction components in brackets)

254x190mm (300 x 300 DPI)

SUPPLEMENTAL APPENDIX

Additional details on the El Cuete cohort

Epidemiological data from the El Cuete IV prospective cohort study were used to inform our findings on syringe exchange program (SEP) utilization. Study details have been described previously [1]. Briefly, eligibility criteria at baseline included injecting drugs in the past month, being at least 18 years old, and not planning to move away from Tijuana over the next 30 months. Participants were recruited by street outreach from March 2011 – May 2013 (N=734), contributing 4,301 study visits in this analysis. Participants were followed biannually, however participants were not required to have injected drugs in the previous six months at their follow-up visits. All participants provided informed consent. The study protocol was approved by the University of California San Diego Human Research Protections Program and El Colegio de la Frontera Norte (Tijuana).

Measures

Our outcome of interest was accessing a SEP (either participants themselves or from someone else who obtained syringes from a SEP) in the past six months. The independent variable of interest was the calendar period, which we classified according to its respective GF period (during and post-GF). The surveys from all visits were sorted in ascending order based on the date of the interview. Next, they were grouped into three-month periods with each period corresponding to a different GF period (during and post-GF). We note that the actual follow-up visits were spaced approximately 6 months apart and the surveys inquired about SEP utilization in the past 6 months, the calendar periods were grouped into three-month periods to allow us to capture and estimate any seasonal variation that might have occurred within each global fund period. For simplicity, in terms of when El Cuete participants may have begun noticing the impact of the GF, we classified the GF period as beginning in March 2012 and ending in June 2014 given that participants were asked about behaviors in the past 6 months. While the dates did

For peer review only

not align exactly with the reported GF start and end times, we assumed that it most likely took the GF sponsored activities a few months to "ramp up" and "wind down". In total, the data were divided into 17 evenly spaced three-month time periods, 9 of which occurred during the GF period and 8 in the post-GF period. On average, there were approximately 200-300 visits per three-month period.

Statistical analyses

We conducted segmented regression, a method used in the evaluation of intervention effects for interrupted time series data [2]. Logistic regression with fixed and random effects was used to estimate the mean log (odds of accessing a SEP) during the previous six months for each of the 17 three-month periods. Next, the mean log odds for each period estimated by the logistic regression model, were used as the outcome variable in a segmented regression analysis, to predict the trend of accessing a SEP within each GF period as well as the level change from the GF period to the post-GF). Given the lack of independent error terms (as the errors of time series data are usually autocorrelated), a linear regression model was fitted to account for the autoregressive error. We tested for autocorrelation using the Breusch-Godfrey test and included first-order and second-order autoregressive terms to adjust for the effect of the positive autocorrelation.

RESULTS

The mean predicted probabilities of accessing a SEP over the 17 three-month periods are shown in Figure S1. Overall, there was a significant increasing trend in the probability of accessing the SEP during the GF period, which peaked in September 2013 (51%, 95% CI: 42% - 59%). During the GF period, the mean log odds of accessing a SEP in the increased by a factor of 0.17 (p-value < 0.001). The level change (the immediate change that occurred between the end of the last three-months of the GF period and the end of the first three months of the post-GF period) from

For peer review only

the GF period to the post-GF period was associated with a 0.73 reduction in the mean log odds of accessing a SEP in the past 6 months (p=0.02). During the post-GF period, the mean log odds of accessing a SEP decreased by a factor of 0.22 (p=0.002).

These trends were roughly consistent with national estimates of syringes acquired with GF support. In 2011, 534,573 syringes were distributed (92,070 financed by the GF [note: some sites may have been receiving funds prior to the SEP in Tijuana]), increasing to 1,904,961 syringes distributed (1,199,520 from the GF) in 2012. In 2013, 3,235,372 syringes were distributed with 78% provided by the GF (2,508,840). By 2014, the number of syringes distributed nationally declined to 643,320 [3].

For peer review only

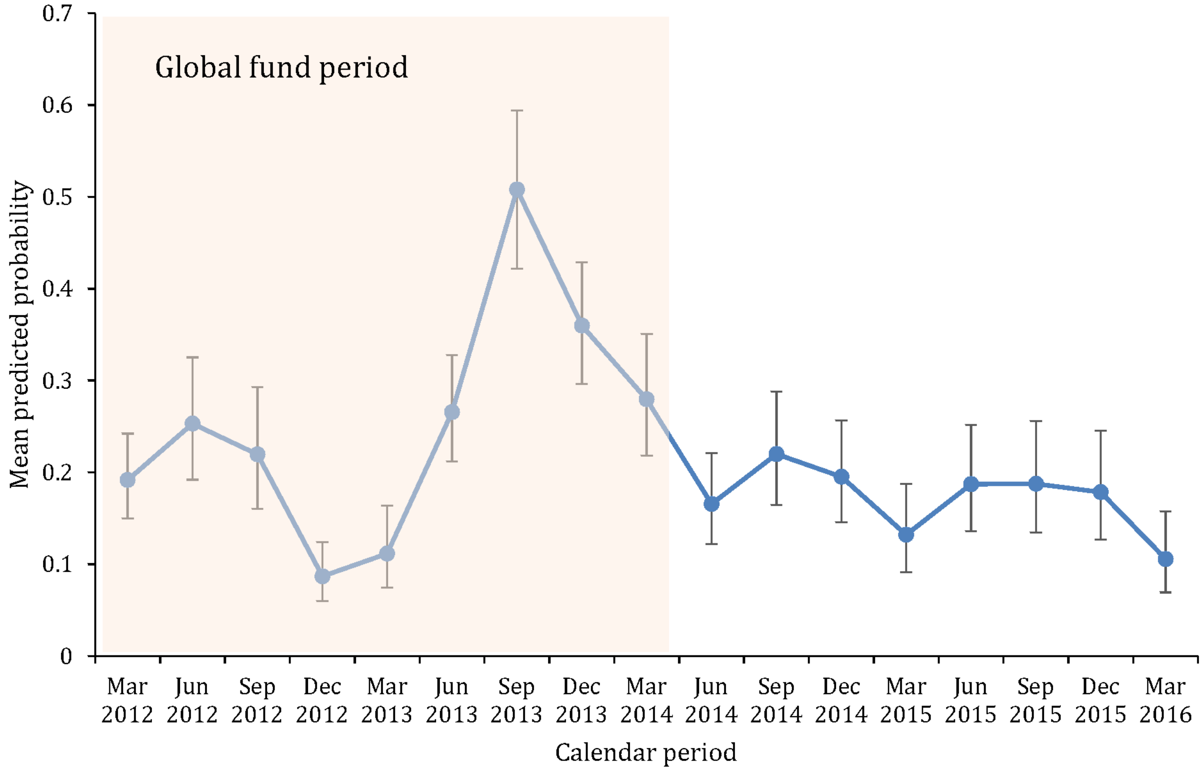

0.7
0.6
0.5
0.4
0.3
0.2
0.1
0
Global fund period
Mean predicted probability
Mar 2012
Jun 2012
Sep 2012
Dec 2012
Mar 2013
Jun 2013
Sep 2013
Dec 2013
Mar 2014
Jun 2014
Sep 2014
Dec 2014
Mar 2015
Jun 2015
Sep 2015
Dec 2015
Mar 2016
Calendar period

Figure S2: Number of syringes distributed in Mexico 2011 – 2014.

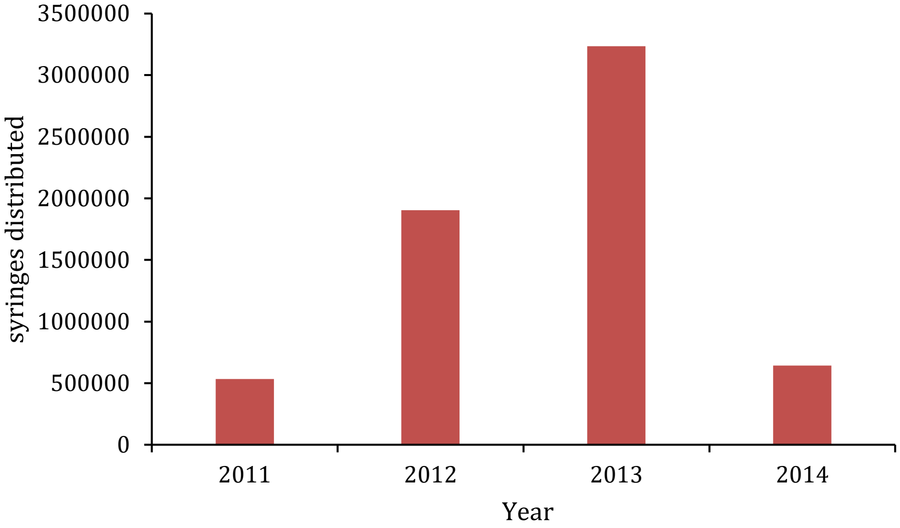

References

1. Robertson AM, Garfein RS, Wagner KD, Mehta SR, Magis-Rodriguez C, Cuevas-Mota J, Moreno-Zuniga PG, Strathdee SA, Proyecto El Cuete IV, Stahr, II. Evaluating the impact of Mexico's drug policy reforms on people who inject drugs in Tijuana, B.C., Mexico, and San Diego, CA, United States: a binational mixed methods research agenda. *Harm Reduct J* 2014, 11:4.
2. Wagner AK, Soumerai SB, Zhang F, Ross-Degnan D. Segmented regression analysis of interrupted time series studies in medication use research. *Journal of clinical pharmacy and therapeutics* 2002, 27(4):299-309.
3. National Report on the Progress of the HIV/AIDS Response (Informe Nacional de Avances en La Respuesta al VIH y el SIDA) [http://www.censida.salud.gob.mx/descargas/ungass/GARPR_Mx2015.pdf]

BMJ Open

Evaluating the impact of Global Fund withdrawal on needle and syringe provision, cost, and use among people who inject drugs in Tijuana, Mexico: a costing analysis

Journal:	*BMJ Open*
Manuscript ID	bmjopen-2018-026298.R1
Article Type:	Research
Date Submitted by the Author:	14-Nov-2018
Complete List of Authors:	Cepeda, Javier; University of California San Diego, Medicine<br>Burgos, Jose Luis; University of California San Diego, Medicine<br>Kahn, James; University of California, San Francisco, Philip R. Lee Institute for Health Policy Studies<br>Padilla, Rosario; Centro de Servicios (SER)<br>Meza, Emilio; Department of Social and Human Development<br>Segovia, Luis Alberto; Prevencasa AC<br>Gaines, Tommi; UCSD,<br>Abramovitz, Daniela; UCSD,<br>Rangel, Gudelia; El Colegio de la Frontera Norte<br>Magis, Carlos; Centro Nacional para la Prevención y el Control del VIH y el Sida (CENSIDA)<br>Vickerman, Peter; University of Bristol, School of Social and Community Medicine<br>Strathdee, Steffanie; University of California San Diego, Medicine<br>Martin, Natasha; University of California, San Diego
**Primary Subject Heading**:	Health services research
Secondary Subject Heading:	HIV/AIDS, Epidemiology
Keywords:	HEALTH ECONOMICS, HIV & AIDS < INFECTIOUS DISEASES, EPIDEMIOLOGY

SCHOLARONE™ Manuscripts

TITLE: Evaluating the impact of Global Fund withdrawal on needle and syringe provision, cost, and use among people who inject drugs in Tijuana, Mexico: a costing analysis

Javier A. Cepeda[1§*], Jose Luis Burgos[1*], James G. Kahn[2], Rosario Padilla[3], Pedro Emilio Meza Martinez[4], Luis Alberto Segovia[5], Tommi Gaines[1], Daniela Abramovitz[1], Gudelia Rangel[6], Carlos Magis-Rodriguez[7], Peter Vickerman[8], Steffanie A. Strathdee[1], Natasha K. Martin[1,8]

1. Division of Infectious Diseases and Global Public Health, 9500 Gilman Drive, MC0507, University of California – San Diego, USA; jacepeda@ucsd.edu, jlburgos@ucsd.edu, togaines@ucsd.edu, dabramovitz@ucsd.edu, sstrathdee@ucsd.edu, natasha-martin@ucsd.edu

2. Institute for Health Policy Studies, School of Medicine, University of California, San Francisco, USA; jgkahn@ucsf.edu

3. Centro de Servicios (SER), Tijuana, Mexico; Gonzalez Ortega 885, Zona Centro, 22000 Tijuana, B.C., Mexico; rosario@centroser.org

4. Department of Social and Human Development, State Government of Oaxaca, Mexico; Privada Yagul 204, Universidad, San Jose la Noria, 68120 Oaxaca, Mexico; emilio.meza@gmail.com

5. Prevencasa A.C., Tijuana, Mexico; Baja California 7580, Zona Centro, 22000, Tijuana, B.C., Mexico luisalberto_segovia@yahoo.com.mx

6. El Colegio de la Frontera Norte, Carretera escénica, Km 18.5, San Antonio del Mar, 22560 Tijuana, B.C., Mexico; grangel2009@gmail.com

7. Centro Nacional para la Prevención y el Control del VIH y el Sida (CENSIDA); Av. Marina Nacional 60 , piso 8, Tacuba, Del. Miguel Hidalgo, 11410, Ciudad de México; carlos.magis@gmail.com

8. School of Social and Community Medicine, University of Bristol, Senate House, Tyndall Avenue, Bristol, BS8 1TH, UK; epxpv@bristol.ac.uk

*contributed equally

§**corresponding author:**

Javier Cepeda
Division of Infectious Diseases and Global Public Health
Department of Medicine
University of California, San Diego
9500 Gilman Drive MC 0507 La Jolla, CA, USA
Ph: 858-534-3197
jacepeda@ucsd.edu

Running head: Cost of needle and syringe program in Mexico

Key words: needle and syringe program, Mexico, Global Fund, cost, harm reduction, people who inject drugs

Word count: 3437

For peer review only - http://bmjopen.bmj.com/site/about/guidelines.xhtml

ABSTRACT

Objective: From 2011 – 2013, the Global Fund supported needle and syringe programs in Mexico to prevent transmission of HIV among people who inject drugs. It remains unclear how Global Fund withdrawal affected the costs, quality, and coverage of needle and syringe program provision.

Design: Costing study and longitudinal cohort study.

Setting: Tijuana, Mexico

Participants: Personnel from a local needle and syringe program (N=6) and people who inject drugs (N=734) participating in a longitudinal study.

Primary outcome measures: Provision of needle and syringe program services and cost (per contact and per syringe distributed, in 2017 $USD) during Global Fund support (2012) and after withdrawal (2015/16). An additional outcome included needle and syringe program utilization from a concurrent cohort of people who inject drugs during and after Global Fund withdrawal.

Results:

During the Global Fund period, the needle and syringe program distributed 55,920 syringes to 932 contacts (60 syringes/contact) across 14 geographical locations. After Global Fund withdrew, the needle and syringe program distributed 10,700 syringes to 2,140 contacts (5 syringes/contact) across 3 geographical locations. During the Global Fund period, the cost per harm reduction contact was approximately 10-fold higher compared to after Global Fund ($44.72 vs. $3.81), however the cost per syringe distributed was nearly equal ($0.75 vs. $0.76) due to differences in syringes per contact and reductions in ancillary kit components. The mean log odds of accessing a needle and syringe program in the post- Global Fund period was significantly lower than during the GF period (p=0.02).

Conclusions:

Withdrawal of Global Fund support for needle and syringe program provision in Mexico was associated with a substantial drop in provision of sterile syringes, geographical coverage, and recent clean syringe utilization among people who inject drugs. Better planning is required to ensure harm reduction program sustainability is at scale after donor withdrawal.

Word count: 300

Strengths and limitations of this study

- We analyzed provision and cost data of a needle and syringe program in Tijuana, Mexico during and after Global Fund withdrawal which we used to estimate how withdrawal impacted quality of the program.
- Our findings were further strengthened with the triangulation of self-reported needle and syringe program utilization data from a concurrent cohort of people who inject drugs in Tijuana.
- We were uncertain about the number of unique clients of the needle and syringe program since only the number of contacts (kits distributed) was provided.

For peer review only - http://bmjopen.bmj.com/site/about/guidelines.xhtml

BACKGROUND

The effectiveness of needle and syringe programs (NSP) in reducing transmission of HIV and hepatitis C virus (HCV) among people who inject drugs (PWID) has been well documented. Findings from a meta-analysis reported that NSPs from higher quality studies were associated with a 58% (95% CI: 0.22 – 0.81) reduction in HIV transmission.[1] Similarly, a recent Cochrane systematic review and meta-analysis found that NSPs were associated with a 23% reduction in HCV transmission (RR=0.79, 95% CI: 0.39 – 1.61), although a stronger effect was seen in Europe (RR=0.24, 95% CI: 0.09-0.62).[2] Despite the protective benefits of these services, the coverage of critical harm reductions services such as NSPs remains suboptimal, especially in low/middle income countries (LMIC)[3] where most of the HIV and HCV disease burden lies.[4]

Program evaluation, such as costing analyses, are important for budgeting and can help policymakers make evidence-based decisions with scarce resources. While LMIC would benefit the most from costing analyses of harm reduction services due to these countries having more limited resources, few economic evaluations of harm reduction services have been published in these settings. Studies conducted in Eastern Europe[5 6], Bangladesh[7], and China[8] showed that harm reduction services can be effective relative to their cost, especially within the context of nascent HIV epidemics among PWID. In Latin America, there have been no economic evaluations of NSPs.

Despite sharing one of the busiest land-border crossings in the world, numerous socioeconomic and health disparities separate Tijuana, Mexico from San Diego, California. Tijuana has a prominent Red-Light district and draws in drug and sex tourists primarily from the United States that has resulted in a localized HIV epidemic.[9] It also has one of the highest concentrations of PWID in Mexico, 4-10% of whom are HIV-infected and >90% of whom are HCV antibody positive.[10 11] NSPs have been operating in Tijuana for more than 15 years, however prevention of transmission remains a challenge. The proportion accessing harm reduction

For peer review only - http://bmjopen.bmj.com/site/about/guidelines.xhtml

services (<10% in the last 6 months in 2011) is lower than the coverage recommended by the WHO[12] who defined "good coverage" as >60% of PWID contacting NSP services at least monthly in the past year.[13]

From 2011 to 2013 the Global Fund (GF) supported NSP provision in Mexico. However, due to Mexico's rising GDP, the GF abruptly withdrew support by December 2013. It is unclear how this withdrawal affected the provision and economics of NSPs in Mexico. Our analysis had two objectives: 1) to compare NSP operations and costs between two periods, in 2012 (when NSPs were receiving funding from the GF) and in 2015 (after GF stopped funding projects in Mexico); and 2) to examine the effect of GF withdrawal on NSP access from PWID enrolled in a longitudinal cohort study in Tijuana. Findings from this analysis may inform harm reduction provision planning and donor support planning in other settings across the region, particularly those who may transition from donor funded to state-funded harm reduction provision.

METHODS

Harm reduction provision and cost data were collected from one NSP site in Tijuana, Mexico. Data collection occurred between March 2016 and February 2017. We examined cost data on NSP provision and cost for two periods: during GF support (2012) and after the GF withdrew support (2015/2016). To estimate provision and costs of an efficient NSP with enhanced resources, we report outcomes during the highest volume month of GF support (May 2012). To estimate current provision and costs of NSP we report average monthly outcomes for 2015/16, and additionally report on provision during the highest volume month of 2015/16 (July 2015) for direct comparison with May 2012.

NSP Characteristics

The NSP was a fixed site located in the Zona Centro (near the "Red-Light" district, a hotspot of illicit drug use and commercial sex activity). Distribution of the number of syringes per contact was reliant upon available funding, however they were provided at no cost to the user. The NSP operated 11 months per year, and

For peer review only - http://bmjopen.bmj.com/site/about/guidelines.xhtml

provided sterile syringes, offered rapid HIV testing/counselling, and referred to hepatitis B and C testing/counselling.

Service provision data collection

During site visits, we reviewed daily logs of geographical outreach of needle and syringe activities, contents of sterile syringe kits, and operating hours. We obtained estimates on the number of contacts and number of syringes distributed per month from activity logs provided by senior staff.

Costing strategy

We costed from an economic perspective, monetizing all input resources, including staff, supplies (purchased or donated), building space, and other items. We used an ingredients-based top-down[14] micro-costing approach where overall inputs were measured at the programmatic level (i.e. we did not observe individual clients or services) separately and combined to generate total and per-client unit costs. We divided total monthly costs by two monthly outputs of interest (1) number of harm reduction contacts and (2) number of sterile syringe kits distributed. The study evaluated current implementation costs and did not consider start-up costs since the NSP has been in operation by a non-governmental organization for several years with support from the federal HIV/AIDS prevention agency in Mexico (CENSIDA).

We classified costs as recurrent (e.g. personnel and non-personnel) and capital. Personnel salaries were obtained from expenditure records, and the number of hours and percent effort dedicated to operating the NSP (including administration) were obtained from interviews with senior staff. During the GF investment period, outreach workers were paid per harm reduction kit distributed. Volunteer costs were calculated based on interviews with senior staff that reported the number of NSP-related hours and the wage a volunteer would have received if they had been employed. Recurrent non-personnel costs included supplies (including syringes and ancillary harm reduction items), building maintenance, utilities, and other services (accounting, maintenance,

For peer review only - http://bmjopen.bmj.com/site/about/guidelines.xhtml

cleaning, security, etc.). Unit prices for inputs were obtained from financial records, itemized bills/receipts, and sales catalogues. Capital costs included building space and equipment. Senior staff provided an overall monthly rent and an estimate of the proportion of building space that was attributed only to provision of NSP services, which we confirmed visually during site visits. We multiplied the rent by the proportion dedicated to NSP services to obtain the operational cost for only NSP provision. All recurrent costs associated with operating a vehicle (fuel, insurance, etc.) were obtained from expenditure records. Equipment and vehicle costs were amortized over the estimated lifespan of the item and then converted into a monthly cost. During the GF period, fuel costs for May 2012 were estimated using daily transportation logs, which were used to calculate miles driven. We used this to convert to estimated litres consumed using estimates of the vehicle's fuel economy multiplied by fuel prices (1 litre = $0.78 USD).[15]

Costing period during Global Fund

We obtained activity logs for May, 2012. Since provision of services varied during the GF period, we intentionally selected this month because it reflected a period of maximum (i.e. "ideal") provision with GF investment according to interviews with study staff which we then contrasted with current levels of provision (post-GF). These personnel costs were inflated to 2017 Mexican pesos using the consumer price index from the Instituto Nacional de Estadistica y Geografia and then converted to US dollars using the January 2017 exchange rate (20.72 Mexican pesos = 1 USD).

Costing period after Global Fund

In addition to cost data during the GF period, we also obtained data after GF withdrawal from 2015/2016 (costing period May – April). Costs were inflated to 2017 Mexican pesos using the midpoint of each cost year, and then converted to USD.

NSP access among PWID living in Tijuana

For peer review only - http://bmjopen.bmj.com/site/about/guidelines.xhtml

Since 2011, members of our research division have followed a cohort of PWID (N=734) living in Tijuana (Project "El Cuete IV") to assess trends and patterns in risk behaviours, HIV incidence, and harm reduction service utilization. Study procedures have been described elsewhere and all participants consented to study procedures.[11] To assess how GF withdrawal may have impacted clean syringe provision among PWID, we analysed data collected from March 2012 – June 2016 (roughly coinciding with the end of the costing period) to determine the proportion of El Cuete participants who reported receiving clean syringes from a NSP within the past 6 months. We applied methods from interrupted time series analysis and conducted segmented regression[16] to estimate significant temporal changes in NSP utilization during the GF period and then after the GF withdrew. We first fit a logistic regression model with fixed and random effects and a first order autoregressive correlation structure to generate the mean predicted probabilities for each quarter of the calendar year. We then fit the mean predicted probabilities into a segmented linear regression model controlling for autoregressive error to estimate the coefficients of accessing a NSP during the different GF periods. Additional details are provided in Additional file 1.

Patient and public involvement

We presented the study to community stakeholders and obtained their approval. Study staff, who had formerly used drugs and reflected the community, reviewed and pre-tested the survey

RESULTS

NSP provision and utilization during Global Fund Period (2012*)*

In 2012, the NSP provided harm reduction services six days per week and offered outreach services which covered a wide geographical area across Tijuana (Figure 1A). Contents of the harm reduction kit included: 60 syringes (all low dead space), 240 alcohol swabs, 20 condoms, 60 3-ml vials of sterile water, 200 cotton swabs, one aluminium sheet, one 60 g tube of lubricant, and one bottle of bleach (Figure 2). In May 2012, the NSP reported 932 harm reduction contacts, resulting in 55,920 syringes distributed (Table 1). Personnel employed

For peer review only - http://bmjopen.bmj.com/site/about/guidelines.xhtml

during this time included: a coordinator, accountant, counsellor, and nurse. Eight health education/outreach workers conducted needle and syringe at various sites and 115 HIV tests were conducted. Based on the monthly activity log, we estimated that 234 km per month were travelled for outreach purposes.

Table 1: Provision of NSP services during the GF period (May, 2012), highest volume (July, 2015), and average month (2015/2016)

	GF-period May 2012	Post-GF period 2015/2016 (highest volume month)	Post-GF period 2015/2016 (average month)
**Unit of service**			
Harm reduction contacts per month	932	3,170	2,140
**Contents of harm reduction kit**			
Syringes	60	5	5
Sterile water	60	3	3
Alcohol swabs	240	5	5
Cotton	200	-	-
Foil	1	-	-
Bleach	1	-	-
Condoms	20	2	2
Lubricant (60 g)	1	-	-
**Syringes provided**	55,920	15,850	10,700
**Number of HIV tests conducted**	115	--†	55

†HIV testing data were not available for the highest volume month

Cost of NSP provision during Global Fund Period (2012)

The total monthly cost of NSP provision in May 2012 was $41,681 (Table 2). The cost per harm reduction contact was $44.72 while the cost per syringe distributed was $0.75. Nearly two-thirds (62%) of the total monthly costs were attributed to ancillary kit components (shown in Figure 2; $26,262) while syringes contributed 27% ($11,395) and 10% consisted of recurrent personnel, non-personnel, and capital costs. The cost of syringe distribution excluding ancillary kit components (personnel + other recurrent + capital + syringes only) during the GF period was $0.28 per syringe distributed.

For peer review only - http://bmjopen.bmj.com/site/about/guidelines.xhtml

Table 2: Capacity, optimum (May 2012) and average monthly costs of NSPs operating in Tijuana, Mexico (all costs in 2017 $USD)

	GF-period May 2012	Post-GF period 2015/2016 (average month)
**Unit of service**		
Harm reduction contacts per month	932	2,140
**Capital cost (monthly)**	**$778**	**$778**
Building/space*	$537	$537
Equipment	$241	$241
**Personnel‡ (monthly)**	**$2,503**	**$2,407**
Coordinators	$748	$748
Accountant	$94	$94
Counselor/Head of harm reduction services	$424	$424
Clinician (Nurse/Physician)	$698	$698
Health educators/Outreach workers	$539	$443
**Non-personnel recurrent costs (monthly)**	**$38,399**	**$4,947**
Syringes	$11,395	$1,853
Supplies	$178	$178
Utilities and other services‡	$564	$485
Ancillary harm reduction contents	$26,262	$2,431
**Total monthly cost§**	**$41,681**	**$8,131**
**Cost per harm reduction contact**	$44.72	$3.80
**Cost per syringe distributed including ancillary kit contents**	$0.75	$0.76
**Cost per syringe distributed excluding ancillary kit contents**	$0.28	$0.53

‡only includes the amount dedicated to providing harm reduction services
§may not sum to total due to rounding

NSP provision and utilization post Global Fund (2015/16)

Operations and provision of harm reduction services differed substantially during and after the GF withdrew support in 2015/16 (Figure 1B and Table 1). As shown in Figure 1B, geographic coverage of providing harm reduction services was sharply reduced after the GF withdrawal and limited mostly to the Zona Norte. Outreach personnel was reduced from 8 workers in 2012 to 4 workers in 2015/2016. Additionally, post-GF, the harm reduction kit contents were substantially reduced compared to the GF period (Table 1 and Figure 2). In 2015, the harm reduction kit contents included five syringes (three low-dead space and two high-dead space, compared to 60 low dead space syringes in 2012), three 3-ml vials of sterile water, five alcohol swabs, and three condoms. Additionally, post-GF, service provision was reduced by one day to five days per week. Despite

For peer review only - http://bmjopen.bmj.com/site/about/guidelines.xhtml

decreased geographic coverage and reduced opening hours, the number of kits distributed per month was higher in 2015 than in 2012. The NSP reported a mean of 2,140 monthly contacts in 2015/16 (3,170 contacts during highest volume month of 2015/16), compared to 932 in May 2012. However, because of the substantial decrease in syringes per kit, the total number of syringes distributed was substantially lower in 2015/16- an average of 10,700 syringes distributed per month in 2015/16. During the highest volume month in 2015/16, there were 15,850 syringes distributed compared to 55,920 in May 2012.

Cost of NSP provision post Global Fund (2015/2016)

The total monthly cost of the NSP was over 5-fold higher during 2012 compared to the average monthly cost during 2015/16 ($41,681 vs. $8,131 respectively), primarily due to the higher cost of the harm reduction kit during the GF period ($44.72 vs. $3.80 per kit, respectively). By comparison, the total monthly cost during the month of maximum provision, post-GF, was $10,193. The cost of just syringe distribution (excluding ancillary kit components) doubled from $0.28/syringe distributed in 2012 to $0.53/syringe distributed in 2015/16, mostly due to the reduction in syringes distributed and thus the higher personnel cost per syringe (Figure 3). However, after including ancillary kit components, there was no change in the cost per syringe distributed in 2015/16 ($0.76) compared to May 2012 ($0.75), because of the reduction in ancillary kit component expenditure. Similarly, there was little change in cost per syringe ($0.64) distributed when comparing to the maximum volume month in 2015/2016. Harm reduction kit (syringes + ancillary components) comprised 90% of the costs per syringe distributed during the GF period, whereas these items comprised only 51% in 2015/2016 (Figure 3).

Temporal trends in NSP access among PWID

Based on data from PWID in the El Cuete IV study, we calculated the mean predicted probabilities of accessing a NSP over the 17 three-month periods, which are shown in Figure S1 (Additional file 1). Overall, there was a significant increasing trend in the probability of accessing the NSP during the GF period, which peaked in September 2013 (51%, 95% CI: 42% - 59%). During the GF period, the mean log odds of accessing a NSP

For peer review only - http://bmjopen.bmj.com/site/about/guidelines.xhtml

increased by a factor of 0.17 (p-value < 0.001). The immediate change that occurred between the end of the last three-months of the GF period and the end of the first three months of the post-GF period was associated with a 0.73 reduction in the mean log odds of accessing a NSP in the past 6 months (p=0.02). During the post-GF period, the mean log odds of accessing a NSP decreased by a factor of 0.22 (p=0.002).

DISCUSSION

Our analysis is among the first to describe the cost of providing needle and syringe services in a Latin American setting and the first to specifically compare coverage and costs of needle and syringe during versus after withdrawal of GF support. We found dramatic declines in geographical coverage and number of syringes and ancillary kit components distributed post-GF withdrawal among one NSP provider in Tijuana, with concomitant declines in reported syringe access among PWID. Excluding ancillary kit components, cost per syringe distributed doubled post-GF; total cost per syringe (including kit components) remained similar across periods as ancillary components were dramatically reduced to cut costs. We expect to use both GF ("ideal") and post-GF ("current") NSP provision costs to inform future cost-effectiveness analyses of NSPs on reducing HIV incidence in LMIC.

Despite dramatic declines in volume, quality, and geographical coverage of NSP post-GF withdrawal, it was encouraging that NSP provision in both periods covered an array of services recommended by the WHO [12]. Among these, the WHO recommends multiple delivery modalities (NSPs operated both fixed sites and mobile outreach), and referral for first aid, drug treatment, voluntary HIV testing and treatment, diagnosis and treatment of STIs. While several of these services are available to NSP clients, evidence based drug treatment programs, such as opioid substitution therapy, have not been scaled up sufficiently in Tijuana.[17] We note that the WHO recommends that harm reduction kits include needles and syringes, condoms, filters, sterile water, swabs, spoons, puncture-proof containers, acidifiers, tourniquets, bleach and other disinfectants, and education material. Many of these items were provided during the GF era, and although this provision was drastically

For peer review only - http://bmjopen.bmj.com/site/about/guidelines.xhtml

reduced post-GF withdrawal, the kits still contained sterile water, alcohol swabs, and condoms. Future provision should emphasize increasing coverage of both needles and syringes as well as ancillary kit components for maximum prevention benefit.

While the longitudinal PWID cohort provided some external validation of our findings on diminished NSP provision after GF withdrawal, some caution is warranted when attempting to triangulate these results. Participants in the study did not specify from which NSP (nor from which geographical site) they received syringes, thus we cannot conclude with any certainty that the participants received their syringes from the NSP that we analysed during and post-GF. Despite this, we still found a highly significant reduced log odds of NSP utilization in the post-GF period. Overall, this finding is consistent with national level data from CENSIDA, which reported an 80% decrease in the number of syringes distributed per PWID from 2013 (19.7 syringes per PWID) to 2014 (3.9 syringes per PWID; see Figure S2).[18]

To our knowledge, our study is the first to estimate the cost of NSP provision in Latin America. Our reported cost per syringe distributed in Tijuana after the GF withdrew support ($0.76 per syringe distributed) is less than estimates in other high-income countries, but higher than other low-middle income country (LMIC) settings. For example, an analysis in the U.S. and Canada estimated a cost of $2.28 per syringe distributed, after inflating to 2017 USD.[19] In the U.K., cost per syringe/needle distributed ranged from $0.28 - $2.17[20] with similar cost per syringe distributed in Australia ($1.94).[21] These differences may be attributed to higher personnel costs which represented 66% of the costs in U.S. and Canadian NSPs, while this comprised approximately 30-40% in Tijuana in the post GF period. Conversely, the cost per harm reduction contact in Mexico was higher than in other LMICs, including Bangladesh ($0.42 in 2017 USD)[7] , China ($0.13 in 2017 USD)[22] and Russia ($0.38 in 2017 USD).[23] Despite a similar number of syringes distributed per month personnel costs were approximately ten times lower in China compared to Mexico in the post GF period. However, we cannot determine whether this was due to greater efficiency or fewer personnel.

For peer review only - http://bmjopen.bmj.com/site/about/guidelines.xhtml

Limitations

Our study has several limitations. First, we did not collect any client cost data so we cannot provide any non-service delivery costs, such as PWID transportation to the NSP site. This distinction may be relevant when comparing 2012 to 2015 since PWID were provided more syringes per harm reduction kit in 2012 and therefore may have not needed to access the NSP as frequently (we saw a correspondingly lower number of contacts per month during this period compared to post-GF). In addition, the geographic reach post-GF was much more limited, potentially substantially increasing transport costs for PWID. We are also uncertain about the number of unique PWID who accessed the NSP during the GF period since we only obtained the number of contacts (kits distributed).

Secondly, we report on changes in NSP provision during and post-GF support from one NSP in Tijuana, however other providers (one additional NSP and pharmacies) also provide sterile syringes.[24] However, interviews with the other NSP provider indicate that the reduction in services is likely generalizable. It is legal to obtain syringes from pharmacies, however PWID often report discrimination and pharmacists' refusal to sell syringes.[25] It is possible that reductions in syringe provision seen within our NSP site due to withdrawal of GF support could have been countered by increase provision of sterile syringes from pharmacies; however, self-reported data from the longitudinal cohort of PWID do not indicate this occurred (data not shown). Unfortunately, the El Cuete IV survey only began collecting information on number of sterile syringes obtained from any source (NSP, pharmacy, etc.) during the last month of Global Fund funding (December 2013) and therefore we were unable to assess individual-level trends before and after. We recognize the importance of collecting these data because previous findings indicate that individual level coverage affects HIV[26] and HCV risk[2 27], and these estimates could provide additional insight on monitoring temporal trends in NSP access and provision. Surveillance of clean syringe provision is critical since NSPs are often supported by donors, which could abruptly withdraw funding.

For peer review only - http://bmjopen.bmj.com/site/about/guidelines.xhtml

CONCLUSIONS

In Tijuana, Mexico, abrupt withdrawal of GF support was associated with dramatic declines in coverage and availability of needle and syringe provision, an effective[28] and cost-effective HIV prevention intervention.[29] NSP provision during the GF period in Tijuana involved greater access to sterile syringes at a similar cost per syringe distributed while also providing abundant ancillary items that promoted safe injecting and sexual practices. The withdrawal of multilateral donors has undoubtedly left an impact on other LMIC and is not unique to Mexico. For example Romania, which lost NSP funding from the Global Fund after joining the EU, saw a precipitous rise in new HIV infections shortly thereafter.[30] In terms of policy implications, it is becoming increasingly important for donors and governments to understand the financial and programmatic implications of abrupt donor withdrawal, and to appropriately plan for transition strategies which ensure that these services are not compromised in terms of quality, coverage, and monetary value.

DECLARATIONS

Ethics approval and consent to participate

Study procedures were approved by the institutional review boards at University of California San Diego Human Research Protections Program and Universidad Autónoma de Baja California and all NSP personnel provided written informed consent. The study protocol for the El Cuete IV was approved by the University of California San Diego Human Research Protections Program and El Colegio de la Frontera Norte (Tijuana).

Consent for publication

Not applicable

Availability of data and material

Costing spreadsheets are available upon reasonable request. El Cuete IV data cannot be shared due to the need to ensure the confidentiality of participants.

For peer review only - http://bmjopen.bmj.com/site/about/guidelines.xhtml

Competing interests

NM has received unrestricted research grants from Gilead unrelated to this work, and honoraria from Gilead and Merck. All other authors declare no competing interests.

Funding

This study was funded by grants from the National Institute on Drug Abuse (R01DA037773, K01DA043421, T32DA023356, R01DA015612), Fogarty International Center (R25TW009343), and by the University of California, San Diego, Center for AIDS Research (CFAR), an NIH-funded program (P30AI036214), which is supported by the following NIH Institutes and Centers: NIAID, NCI, NIMH, NIDA, NICHD, NHLBI, NIA, NIGMS, and NIDDK.

Authors' contributions

JC, JB, SS and NM conceived and designed the analysis and contributed to the writing of the manuscript. JC and JB conducted the analyses with input from JK, DA, TG, and PV. JB and PM collected cost data with assistance from RP, LAS, GR, and CM. All authors critically reviewed and approved the final version.

Acknowledgements

We thank the staff from the NSP and all respondents from the El Cuete IV study for participating.

For peer review only

For peer review only - http://bmjopen.bmj.com/site/about/guidelines.xhtml

REFERENCES

1. Aspinall EJ, Nambiar D, Goldberg DJ, et al. Are needle and syringe programmes associated with a reduction in HIV transmission among people who inject drugs: a systematic review and meta-analysis. *International journal of epidemiology* 2014;43(1):235-48. doi: 10.1093/ije/dyt243
2. Platt L, Minozzi S, Reed J, et al. Needle syringe programmes and opioid substitution therapy for preventing hepatitis C transmission in people who inject drugs. *The Cochrane database of systematic reviews* 2017;9:Cd012021. doi: 10.1002/14651858.CD012021.pub2 [published Online First: 2017/09/19]
3. Harm Reduction International. The Global State of Harm Reduction 2016, 2016.
4. Mathers BM, Degenhardt L, Phillips B, et al. Global epidemiology of injecting drug use and HIV among people who inject drugs: a systematic review. *The Lancet* 2008;372(9651):1733-45.
5. Kumaranayake L, Vickerman P, Walker D, et al. The cost-effectiveness of HIV preventive measures among injecting drug users in Svetlogorsk, Belarus. *Addiction* 2004;99(12):1565-76. doi: 10.1111/j.1360-0443.2004.00899.x
6. Vickerman P, Kumaranayake L, Balakireva O, et al. The cost-effectiveness of expanding harm reduction activities for injecting drug users in Odessa, Ukraine. *Sexually transmitted diseases* 2006;33(10 Suppl):S89-102. doi: 10.1097/01.olq.0000221335.80508.fa
7. Guinness L, Vickerman P, Quayyum Z, et al. The cost-effectiveness of consistent and early intervention of harm reduction for injecting drug users in Bangladesh. *Addiction* 2010;105(2):319-28. doi: 10.1111/j.1360-0443.2009.02755.x
8. Zhang L, Yap L, Xun Z, et al. Needle and syringe programs in Yunnan, China yield health and financial return. *BMC public health* 2011;11:250. doi: 10.1186/1471-2458-11-250
9. Mehta SR, Wertheim JO, Brouwer KC, et al. HIV transmission networks in the San Diego–Tijuana border region. *EBioMedicine* 2015;2(10):1456-63.
10. White EF, Garfein RS, Brouwer KC, et al. Prevalence of hepatitis C virus and HIV infection among injection drug users in two Mexican cities bordering the US. *Salud Publica Mexico* 2007;49(3):165-72. doi: Doi 10.1590/S0036-36342007000300001
11. Robertson AM, Garfein RS, Wagner KD, et al. Evaluating the impact of Mexico's drug policy reforms on people who inject drugs in Tijuana, B.C., Mexico, and San Diego, CA, United States: a binational mixed methods research agenda. *Harm Reduct J* 2014;11:4. doi: 10.1186/1477-7517-11-4
12. World Health Organization. Guide to starting and managing needle and syringe programmes. 2007
13. WHO and UNODC. Technical Guide for countries to set targets for universal access to HIV prevention, treatment and care for injecting drug users. 2009
14. Cunnama L, Sinanovic E, Ramma L, et al. Using Top-down and Bottom-up Costing Approaches in LMICs: The Case for Using Both to Assess the Incremental Costs of New Technologies at Scale. *Health Economics* 2016;25:53-66. doi: 10.1002/hec.3295
15. Dibble S. Gas price protest leads to shortages at Tijuana stations San Diego Union Tribune2017 [Available from: http://www.sandiegouniontribune.com/news/border-baja-california/sd-me-gas-shortage-tjuana-20170106-story.html accessed 12 April 2017.
16. Wagner AK, Soumerai SB, Zhang F, et al. Segmented regression analysis of interrupted time series studies in medication use research. *Journal of clinical pharmacy and therapeutics* 2002;27(4):299-309.
17. Werb D, Wagner K, Beletsky L, et al. Police bribery and access to methadone maintenance therapy within the context of drug policy reform in Tijuana, Mexico. *Drug Alcohol Depen* 2015;148:221-25.
18. CENSIDA. National Report on the Progress of the HIV/AIDS Response (Informe Nacional de Avances en La Respuesta al VIH y el SIDA) 2015 [Available from: http://www.censida.salud.gob.mx/descargas/ungass/GARPR_Mx2015.pdf accessed 25 May 2018.
19. Lurie P, Reingold AL. The Public Health Impact of Needle Exchange Programs in the United States and Abroad: Summary, Conclusions, and Recommendations. 1993
20. Platt L, Sweeney S, Ward Z, et al. Assessing the impact and cost-effectiveness of needle and syringe provision and opioid substitution therapy on hepatitis C transmission among people who inject drugs in the UK: an analysis of pooled data sets and economic modelling. 2017
21. Wilson D, Kwon A, Anderson J, et al. Return on investment 2: Evaluating the cost-effectiveness of needle and syringe programs in Australia. *Sydney: National Centre in HIV Epidemiology and Clinical Research, The University of New South Wales* 2009

For peer review only - http://bmjopen.bmj.com/site/about/guidelines.xhtml

22. Masaki E. CJ, Zhu Q., Chen X., Chen Y., Lu M. Cost -Effectiveness of Harm Reduction Interventions in Guangxi Zhuang Autonomous Region, China. *World Bank* 2007
23. Marseille E, Dandona L, Saba J, et al. Assessing the efficiency of HIV prevention around the world: methods of the PANCEA project. *Health services research* 2004;39(6p2):1993-2012.
24. Pollini RA, Lozada R, Gallardo M, et al. Barriers to Pharmacy-Based Syringe Purchase Among Injection Drug Users in Tijuana, Mexico: A Mixed Methods Study. *Aids Behav* 2010;14(3):679-87. doi: 10.1007/s10461-010-9674-3
25. Davidson PJ, Lozada R, Rosen PC, et al. Negotiating access: social barriers to purchasing syringes at pharmacies in Tijuana, Mexico. *Int J Drug Policy* 2012;23(4):286-94. doi: 10.1016/j.drugpo.2012.05.001
26. Bluthenthal RN, Anderson R, Flynn NM, et al. Higher syringe coverage is associated with lower odds of HIV risk and does not increase unsafe syringe disposal among syringe exchange program clients. *Drug Alcohol Depen* 2007;89(2-3):214-22.
27. Turner KM, Hutchinson S, Vickerman P, et al. The impact of needle and syringe provision and opiate substitution therapy on the incidence of hepatitis C virus in injecting drug users: pooling of UK evidence. *Addiction* 2011;106(11):1978-88.
28. Hurley SF, Jolley DJ, Kaldor JM. Effectiveness of needle-exchange programmes for prevention of HIV infection. *The Lancet* 1997;349(9068):1797-800.
29. Wilson DP, Donald B, Shattock AJ, et al. The cost-effectiveness of harm reduction. *Int J Drug Policy* 2015;26 Suppl 1:S5-11. doi: 10.1016/j.drugpo.2014.11.007
30. Csete J, Kamarulzaman A, Kazatchkine M, et al. Public health and international drug policy. *Lancet* 2016;387(10026):1427-80. doi: 10.1016/S0140-6736(16)00619-X [published Online First: 2016/03/30]

For peer review only

For peer review only - http://bmjopen.bmj.com/site/about/guidelines.xhtml

FIGURES

Figure 1: Geographical coverage of needle and syringe program outreach sites (a) during GF period in May 2012 and (b) after GF withdrawal in 2015/16.

Figure 2: Harm reduction kit components per sterile syringe distributed during the Global Fund Period and after withdrawal of Global Fund. Note: Some items are only partially depicted since more syringes per item were distributed (e.g. during the GF period, three syringes were distributed per one condom).

Figure 3: Cost breakdown per syringe distributed at the NSP site (ancillary harm reduction components in brackets).

For peer review only

For peer review only - http://bmjopen.bmj.com/site/about/guidelines.xhtml

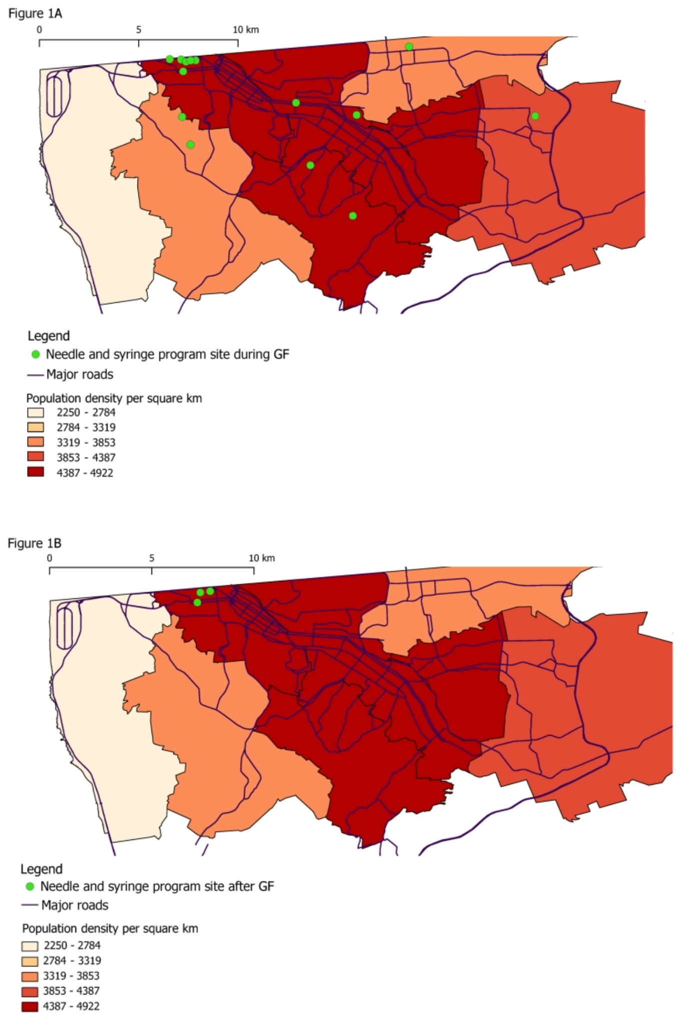

Figure 1: Geographical coverage of needle and syringe program outreach sites (a) during GF period in May 2012 and (b) after GF withdrawal in 2015/16.

190x338mm (300 x 300 DPI)

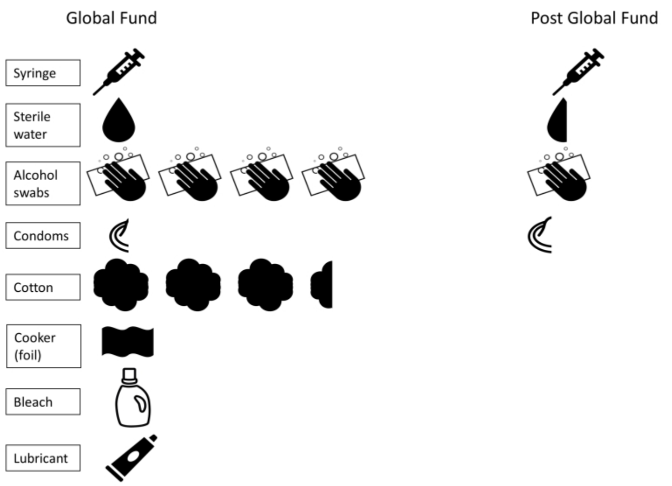

Figure 2: Harm reduction kit components per sterile syringe distributed during the Global Fund Period and after withdrawal of Global Fund. Note: Some items are only partially depicted since more syringes per item were distributed (e.g. during the GF period, three syringes were distributed per one condom).

338x190mm (300 x 300 DPI)

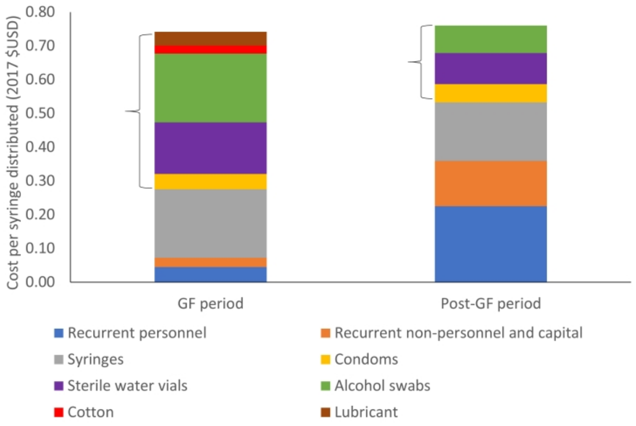

Figure 3: Cost breakdown per syringe distributed at the NSP site (ancillary harm reduction components in brackets).

338x190mm (300 x 300 DPI)

SUPPLEMENTAL APPENDIX

Additional details on the El Cuete cohort

Epidemiological data from the El Cuete IV prospective cohort study were used to inform our findings on needle and syringe program (NSP) utilization. Study details have been described previously.[1] Briefly, eligibility criteria at baseline included injecting drugs in the past month, being at least 18 years old, and not planning to move away from Tijuana over the next 30 months. Participants were recruited by street outreach from March 2011 – May 2013 (N=734), contributing 4,301 study visits in this analysis. Participants were followed biannually, however participants were not required to have injected drugs in the previous six months at their follow-up visits. All participants provided informed consent. The study protocol was approved by the University of California San Diego Human Research Protections Program and El Colegio de la Frontera Norte (Tijuana).

Measures

Our outcome of interest was accessing a NSP (either participants themselves or from someone else who obtained syringes from a NSP) in the past six months. The independent variable of interest was the calendar period, which we classified according to its respective GF period (during and post-GF). The surveys from all visits were sorted in ascending order based on the date of the interview. Next, they were grouped into three-month periods with each period corresponding to a different GF period (during and post-GF). We note that while the actual follow-up visits were spaced approximately 6 months apart and the surveys inquired about NSP utilization in the past 6 months, the calendar periods were grouped into three-month periods to allow us to capture and estimate any seasonal variation that might have occurred within each GF period. For simplicity, in terms of when El Cuete participants may have begun noticing the impact of the GF, we classified the GF period as beginning in March 2012 and ending before June 2014 given that participants were asked about behaviors in the past 6 months. While the

For peer review only - http://bmjopen.bmj.com/site/about/guidelines.xhtml

dates did not align exactly with the reported GF start and end times, we assumed that it most likely took the GF sponsored activities a few months to “ramp up” and “wind down”. For example, prevention programs did not begin to be implemented in Tijuana until the third trimester of 2011.[2] In total, the data were divided into 17 evenly spaced three-month time periods, 9 of which occurred during the GF period and 8 in the post-GF period. On average, there were approximately 200-300 visits per three-month period.

Statistical analyses

We conducted segmented regression, a method used in the evaluation of intervention effects for interrupted time series data.[3] Logistic regression with fixed and random effects was used to estimate the mean log (odds of accessing a NSP) during the previous six months for each of the 17 three-month periods. Next, the mean log odds for each period estimated by the logistic regression model were used as the outcome variable in a segmented regression analysis, to predict the trend of accessing a NSP within each GF period as well as the level change from the GF period to the post-GF). Given the lack of independent error terms (as the errors of time series data are usually autocorrelated), a linear regression model was fitted to account for the autoregressive error. We tested for autocorrelation using the Breusch-Godfrey test and included first-order and second-order autoregressive terms to adjust for the effect of the positive autocorrelation.

RESULTS

The mean predicted probabilities of accessing a NSP over the 17 three-month periods are shown in Figure S1. Overall, there was a significant increasing trend in the probability of accessing the NSP during the GF period, which peaked in September 2013 (51%, 95% CI: 42% - 59%). During the GF period, the mean log odds of accessing a NSP in the increased by a factor of 0.17 (p-value < 0.001). The level change (the immediate change that occurred between the end of the last

For peer review only - http://bmjopen.bmj.com/site/about/guidelines.xhtml

three-months of the GF period and the end of the first three months of the post-GF period) from the GF period to the post-GF period was associated with a 0.73 reduction in the mean log odds of accessing a NSP in the past 6 months (p=0.02). During the post-GF period, the mean log odds of accessing a NSP decreased by a factor of 0.22 (p=0.002).

These trends were roughly consistent with national estimates of syringes acquired with GF support. In 2011, 534,573 syringes were distributed (92,070 financed by the GF [note: some sites may have been receiving funds prior to the NSP in Tijuana]), increasing to 1,904,961 syringes distributed (1,199,520 from the GF) in 2012. In 2013, 3,235,372 syringes were distributed with 78% provided by the GF (2,508,840). By 2014, the number of syringes distributed nationally declined to 643,320.[4]

For peer review only - http://bmjopen.bmj.com/site/about/guidelines.xhtml

Figure S1: Mean predicted probability of accessing a syringe exchange program in past six months among PWID in Tijuana. Error bars represent 95% confidence intervals. Shaded box represents the calendar period when the Global Fund operated in Mexico.

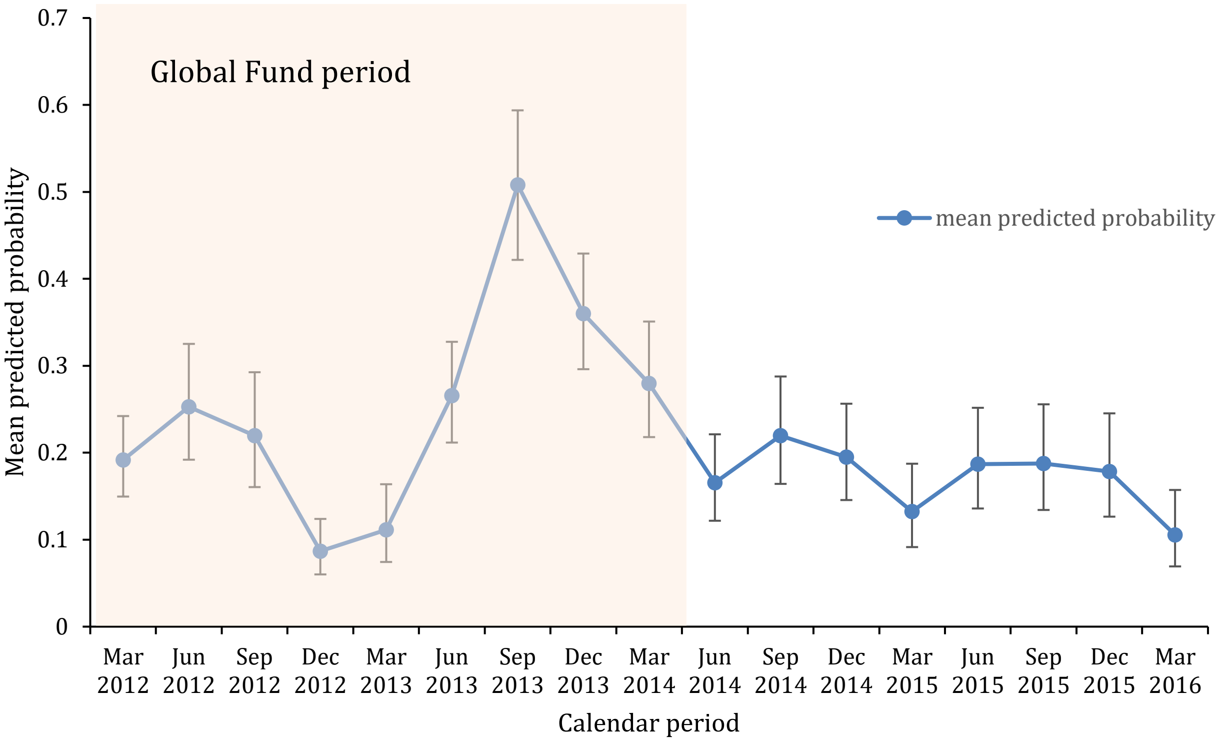

For peer review only - http://bmjopen.bmj.com/site/about/guidelines.xhtml

Figure S2: Number of syringes distributed in Mexico 2011 – 2014[4]

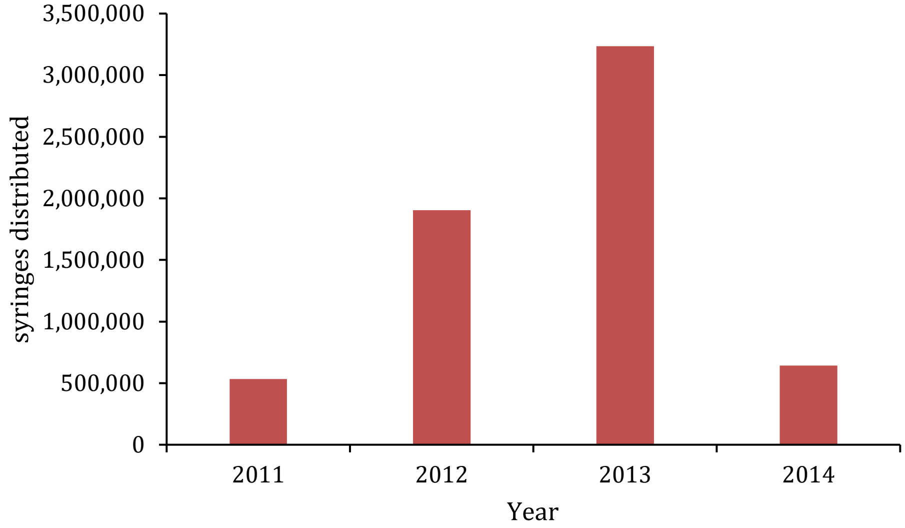

For peer review only

For peer review only - http://bmjopen.bmj.com/site/about/guidelines.xhtml

References

1. Robertson AM, Garfein RS, Wagner KD, et al. Evaluating the impact of Mexico's drug policy reforms on people who inject drugs in Tijuana, B.C., Mexico, and San Diego, CA, United States: a binational mixed methods research agenda. *Harm Reduct J.* 2014;11:4.
2. Fundación Mexicana para la Salud. "Fortalecimiento de las estrategias nacionales de prevención y reducción de daños dirigidos a HSH, HSH/TS, y UDI de ambos sexos". Plan de Implementación. 2011; http://funsalud.org.mx/vihfmfms/aprobados_fm/MX_Plan_Implentacion.pdf. Accessed 24 October, 2018.
3. Wagner AK, Soumerai SB, Zhang F, Ross-Degnan D. Segmented regression analysis of interrupted time series studies in medication use research. *Journal of clinical pharmacy and therapeutics.* 2002;27(4):299-309.
4. CENSIDA. National Report on the Progress of the HIV/AIDS Response (Informe Nacional de Avances en La Respuesta al VIH y el SIDA). 2015; http://www.censida.salud.gob.mx/descargas/ungass/GARPR_Mx2015.pdf. Accessed 25 May, 2018.

For peer review only

BMJ Open

Evaluating the impact of Global Fund withdrawal on needle and syringe provision, cost, and use among people who inject drugs in Tijuana, Mexico: a costing analysis

Journal:	*BMJ Open*
Manuscript ID	bmjopen-2018-026298.R2
Article Type:	Research
Date Submitted by the Author:	13-Dec-2018
Complete List of Authors:	Cepeda, Javier; University of California San Diego, Medicine<br>Burgos, Jose Luis; University of California San Diego, Medicine<br>Kahn, James; University of California, San Francisco, Philip R. Lee Institute for Health Policy Studies<br>Padilla, Rosario; Centro de Servicios (SER)<br>Meza, Emilio; Department of Social and Human Development<br>Segovia, Luis Alberto; Prevencasa AC<br>Gaines, Tommi; UCSD,<br>Abramovitz, Daniela; UCSD,<br>Rangel, Gudelia; El Colegio de la Frontera Norte<br>Magis, Carlos; Centro Nacional para la Prevención y el Control del VIH y el Sida (CENSIDA)<br>Vickerman, Peter; University of Bristol, School of Social and Community Medicine<br>Strathdee, Steffanie; University of California San Diego, Medicine<br>Martin, Natasha; University of California, San Diego
**Primary Subject Heading**:	Health services research
Secondary Subject Heading:	HIV/AIDS, Epidemiology
Keywords:	HEALTH ECONOMICS, HIV & AIDS < INFECTIOUS DISEASES, EPIDEMIOLOGY

SCHOLARONE™
Manuscripts

TITLE: Evaluating the impact of Global Fund withdrawal on needle and syringe provision, cost, and use among people who inject drugs in Tijuana, Mexico: a costing analysis

Javier A. Cepeda[1§*], Jose Luis Burgos[1*], James G. Kahn[2], Rosario Padilla[3], Pedro Emilio Meza Martinez[4], Luis Alberto Segovia[5], Tommi Gaines[1], Daniela Abramovitz[1], Gudelia Rangel[6], Carlos Magis-Rodriguez[7], Peter Vickerman[8], Steffanie A. Strathdee[1], Natasha K. Martin[1,8]

1. Division of Infectious Diseases and Global Public Health, 9500 Gilman Drive, MC0507, University of California – San Diego, USA; jacepeda@ucsd.edu, jlburgos@ucsd.edu, togaines@ucsd.edu, dabramovitz@ucsd.edu, sstrathdee@ucsd.edu, natasha-martin@ucsd.edu

2. Institute for Health Policy Studies, School of Medicine, University of California, San Francisco, USA; jgkahn@ucsf.edu

3. Centro de Servicios (SER), Tijuana, Mexico; Gonzalez Ortega 885, Zona Centro, 22000 Tijuana, B.C., Mexico; rosario@centroser.org

4. Department of Social and Human Development, State Government of Oaxaca, Mexico; Privada Yagul 204, Universidad, San Jose la Noria, 68120 Oaxaca, Mexico; emilio.meza@gmail.com

5. Prevencasa A.C., Tijuana, Mexico; Baja California 7580, Zona Centro, 22000, Tijuana, B.C., Mexico luisalberto_segovia@yahoo.com.mx

6. El Colegio de la Frontera Norte, Carretera escénica, Km 18.5, San Antonio del Mar, 22560 Tijuana, B.C., Mexico; grangel2009@gmail.com

7. Centro Nacional para la Prevención y el Control del VIH y el Sida (CENSIDA); Av. Marina Nacional 60 , piso 8, Tacuba, Del. Miguel Hidalgo, 11410, Ciudad de México; carlos.magis@gmail.com

8. School of Social and Community Medicine, University of Bristol, Senate House, Tyndall Avenue, Bristol, BS8 1TH, UK; epxpv@bristol.ac.uk

*contributed equally

§**corresponding author:**

Javier Cepeda
Division of Infectious Diseases and Global Public Health
Department of Medicine
University of California, San Diego
9500 Gilman Drive MC 0507 La Jolla, CA, USA
Ph: 858-534-3197
jacepeda@ucsd.edu

Running head: Cost of needle and syringe program in Mexico

Key words: needle and syringe program, Mexico, Global Fund, cost, harm reduction, people who inject drugs

Word count: 3437

For peer review only

ABSTRACT

Objective: From 2011 – 2013, the Global Fund supported needle and syringe programs in Mexico to prevent transmission of HIV among people who inject drugs. It remains unclear how Global Fund withdrawal affected the costs, quality, and coverage of needle and syringe program provision.

Design: Costing study and longitudinal cohort study.

Setting: Tijuana, Mexico

Participants: Personnel from a local needle and syringe program (N=6) and people who inject drugs (N=734) participating in a longitudinal study.

Primary outcome measures: Provision of needle and syringe program services and cost (per contact and per syringe distributed, in 2017 $USD) during Global Fund support (2012) and after withdrawal (2015/16). An additional outcome included needle and syringe program utilization from a concurrent cohort of people who inject drugs during and after Global Fund withdrawal.

Results:

During the Global Fund period, the needle and syringe program distributed 55,920 syringes to 932 contacts (60 syringes/contact) across 14 geographical locations. After Global Fund withdrew, the needle and syringe program distributed 10,700 syringes to 2,140 contacts (5 syringes/contact) across 3 geographical locations. During the Global Fund period, the cost per harm reduction contact was approximately 10-fold higher compared to after Global Fund ($44.72 vs. $3.81), however the cost per syringe distributed was nearly equal ($0.75 vs. $0.76) due to differences in syringes per contact and reductions in ancillary kit components. The mean log odds of accessing a needle and syringe program in the post- Global Fund period was significantly lower than during the GF period (p=0.02).

Conclusions:

Withdrawal of Global Fund support for needle and syringe program provision in Mexico was associated with a substantial drop in provision of sterile syringes, geographical coverage, and recent clean syringe utilization among people who inject drugs. Better planning is required to ensure harm reduction program sustainability is at scale after donor withdrawal.

Word count: 300

Strengths and limitations of this study

- We analyzed provision and cost data of a needle and syringe program in Tijuana, Mexico during and after Global Fund withdrawal which we used to estimate how withdrawal impacted quality of the program.
- Our findings were further strengthened with the triangulation of self-reported needle and syringe program utilization data from a concurrent cohort of people who inject drugs in Tijuana.
- We were uncertain about the number of unique clients of the needle and syringe program since only the number of contacts (kits distributed) was provided.

For peer review only - http://bmjopen.bmj.com/site/about/guidelines.xhtml

BACKGROUND

The effectiveness of needle and syringe programs (NSP) in reducing transmission of HIV and hepatitis C virus (HCV) among people who inject drugs (PWID) has been well documented. Findings from a meta-analysis reported that NSPs from higher quality studies were associated with a 58% (95% CI: 0.22 – 0.81) reduction in HIV transmission.[1] Similarly, a recent Cochrane systematic review and meta-analysis found that NSPs were associated with a 23% reduction in HCV transmission (RR=0.79, 95% CI: 0.39 – 1.61), although a stronger effect was seen in Europe (RR=0.24, 95% CI: 0.09-0.62).[2] Despite the protective benefits of these services, the coverage of critical harm reductions services such as NSPs remains suboptimal, especially in low/middle income countries (LMIC)[3] where most of the HIV and HCV disease burden lies.[4]

Program evaluation, such as costing analyses, are important for budgeting and can help policymakers make evidence-based decisions with scarce resources. While LMIC would benefit the most from costing analyses of harm reduction services due to these countries having more limited resources, few economic evaluations of harm reduction services have been published in these settings. Studies conducted in Eastern Europe[5 6], Bangladesh[7], and China[8] showed that harm reduction services can be effective relative to their cost, especially within the context of nascent HIV epidemics among PWID. In Latin America, there have been no economic evaluations of NSPs.

Despite sharing one of the busiest land-border crossings in the world, numerous socioeconomic and health disparities separate Tijuana, Mexico from San Diego, California. Tijuana has a prominent Red-Light district and draws in drug and sex tourists primarily from the United States that has resulted in a localized HIV epidemic.[9] It also has one of the highest concentrations of PWID in Mexico, 4-10% of whom are HIV-infected and >90% of whom are HCV antibody positive.[10 11] NSPs have been operating in Tijuana for more than 15 years, however prevention of transmission remains a challenge. The proportion accessing harm reduction

For peer review only - http://bmjopen.bmj.com/site/about/guidelines.xhtml

services (<10% in the last 6 months in 2011) is lower than the coverage recommended by the WHO[12] who defined "good coverage" as >60% of PWID contacting NSP services at least monthly in the past year.[13]

From 2011 to 2013 the Global Fund (GF) supported NSP provision in Mexico. However, due to Mexico's rising GDP, the GF abruptly withdrew support by December 2013. It is unclear how this withdrawal affected the provision and economics of NSPs in Mexico. Our analysis had two objectives: 1) to compare NSP operations and costs between two periods, in 2012 (when NSPs were receiving funding from the GF) and in 2015 (after GF stopped funding projects in Mexico); and 2) to examine the effect of GF withdrawal on NSP access from PWID enrolled in a longitudinal cohort study in Tijuana. Findings from this analysis may inform harm reduction provision planning and donor support planning in other settings across the region, particularly those who may transition from donor funded to state-funded harm reduction provision.

METHODS

Harm reduction provision and cost data were collected from one NSP site in Tijuana, Mexico. Data collection occurred between March 2016 and February 2017. We examined cost data on NSP provision and cost for two periods: during GF support (2012) and after the GF withdrew support (2015/2016). To estimate provision and costs of an efficient NSP with enhanced resources, we report outcomes during the highest volume month of GF support (May 2012). To estimate current provision and costs of NSP we report average monthly outcomes for 2015/16, and additionally report on provision during the highest volume month of 2015/16 (July 2015) for direct comparison with May 2012.

NSP Characteristics

The NSP was a fixed site located in the Zona Centro (near the "Red-Light" district, a hotspot of illicit drug use and commercial sex activity). Distribution of the number of syringes per contact was reliant upon available funding, however they were provided at no cost to the user. The NSP operated 11 months per year, and

For peer review only - http://bmjopen.bmj.com/site/about/guidelines.xhtml

provided sterile syringes, offered rapid HIV testing/counselling, and referred to hepatitis B and C testing/counselling.

Service provision data collection

During site visits, we reviewed daily logs of geographical outreach of needle and syringe activities, contents of sterile syringe kits, and operating hours. We obtained estimates on the number of contacts and number of syringes distributed per month from activity logs provided by senior staff.

Costing strategy

We costed from an economic perspective, monetizing all input resources, including staff, supplies (purchased or donated), building space, and other items. We used an ingredients-based top-down[14] micro-costing approach where overall inputs were measured at the programmatic level (i.e. we did not observe individual clients or services) separately and combined to generate total and per-client unit costs. We divided total monthly costs by two monthly outputs of interest (1) number of harm reduction contacts and (2) number of sterile syringe kits distributed. The study evaluated current implementation costs and did not consider start-up costs since the NSP has been in operation by a non-governmental organization for several years with support from the federal HIV/AIDS prevention agency in Mexico (CENSIDA).

We classified costs as recurrent (e.g. personnel and non-personnel) and capital. Personnel salaries were obtained from expenditure records, and the number of hours and percent effort dedicated to operating the NSP (including administration) were obtained from interviews with senior staff. During the GF investment period, outreach workers were paid per harm reduction kit distributed. Volunteer costs were calculated based on interviews with senior staff that reported the number of NSP-related hours and the wage a volunteer would have received if they had been employed. Recurrent non-personnel costs included supplies (including syringes and ancillary harm reduction items), building maintenance, utilities, and other services (accounting, maintenance,

For peer review only - http://bmjopen.bmj.com/site/about/guidelines.xhtml

cleaning, security, etc.). Unit prices for inputs were obtained from financial records, itemized bills/receipts, and sales catalogues. Capital costs included building space and equipment. Senior staff provided an overall monthly rent and an estimate of the proportion of building space that was attributed only to provision of NSP services, which we confirmed visually during site visits. We multiplied the rent by the proportion dedicated to NSP services to obtain the operational cost for only NSP provision. All recurrent costs associated with operating a vehicle (fuel, insurance, etc.) were obtained from expenditure records. Equipment and vehicle costs were amortized over the estimated lifespan of the item and then converted into a monthly cost. During the GF period, fuel costs for May 2012 were estimated using daily transportation logs, which were used to calculate miles driven. We used this to convert to estimated litres consumed using estimates of the vehicle's fuel economy multiplied by fuel prices (1 litre = $0.78 USD).[15]

Costing period during Global Fund

We obtained activity logs for May, 2012. Since provision of services varied during the GF period, we intentionally selected this month because it reflected a period of maximum (i.e. "ideal") provision with GF investment according to interviews with study staff which we then contrasted with current levels of provision (post-GF). These personnel costs were inflated to 2017 Mexican pesos using the consumer price index from the Instituto Nacional de Estadistica y Geografia and then converted to US dollars using the January 2017 exchange rate (20.72 Mexican pesos = 1 USD).

Costing period after Global Fund

In addition to cost data during the GF period, we also obtained data after GF withdrawal from 2015/2016 (costing period May – April). Costs were inflated to 2017 Mexican pesos using the midpoint of each cost year, and then converted to USD.

NSP access among PWID living in Tijuana

For peer review only - http://bmjopen.bmj.com/site/about/guidelines.xhtml

Since 2011, members of our research division have followed a cohort of PWID (N=734) living in Tijuana (Project "El Cuete IV") to assess trends and patterns in risk behaviours, HIV incidence, and harm reduction service utilization. Study procedures have been described elsewhere and all participants consented to study procedures.[11] To assess how GF withdrawal may have impacted clean syringe provision among PWID, we analysed data collected from March 2012 – June 2016 (roughly coinciding with the end of the costing period) to determine the proportion of El Cuete participants who reported receiving clean syringes from a NSP within the past 6 months. We applied methods from interrupted time series analysis and conducted segmented regression[16] to estimate significant temporal changes in NSP utilization during the GF period and then after the GF withdrew. We first fit a logistic regression model with fixed and random effects and a first order autoregressive correlation structure to generate the mean predicted probabilities for each quarter of the calendar year. We then fit the mean predicted probabilities into a segmented linear regression model controlling for autoregressive error to estimate the coefficients of accessing a NSP during the different GF periods. Additional details are provided in Additional file 1.

Patient and public involvement

We presented the study to community stakeholders and obtained their approval. Study staff, who had formerly used drugs and reflected the community, reviewed and pre-tested the survey

RESULTS

NSP provision and utilization during Global Fund Period (2012*)*

In 2012, the NSP provided harm reduction services six days per week and offered outreach services which covered a wide geographical area across Tijuana (Figure 1A). Contents of the harm reduction kit included: 60 syringes (all low dead space), 240 alcohol swabs, 20 condoms, 60 3-ml vials of sterile water, 200 cotton swabs, one aluminium sheet, one 60 g tube of lubricant, and one bottle of bleach (Figure 2). In May 2012, the NSP reported 932 harm reduction contacts, resulting in 55,920 syringes distributed (Table 1). Personnel employed

For peer review only - http://bmjopen.bmj.com/site/about/guidelines.xhtml

during this time included: a coordinator, accountant, counsellor, and nurse. Eight health education/outreach workers conducted needle and syringe at various sites and 115 HIV tests were conducted. Based on the monthly activity log, we estimated that 234 km per month were travelled for outreach purposes.

Table 1: Provision of NSP services during the GF period (May, 2012), highest volume (July, 2015), and average month (2015/2016)

	GF-period May 2012	Post-GF period 2015/2016 (highest volume month)	Post-GF period 2015/2016 (average month)
**Unit of service**			
Harm reduction contacts per month	932	3,170	2,140
**Contents of harm reduction kit**			
Syringes	60	5	5
Sterile water	60	3	3
Alcohol swabs	240	5	5
Cotton	200	-	-
Foil	1	-	-
Bleach	1	-	-
Condoms	20	2	2
Lubricant (60 g)	1	-	-
**Syringes provided**	55,920	15,850	10,700
**Number of HIV tests conducted**	115	--†	55

†HIV testing data were not available for the highest volume month

Cost of NSP provision during Global Fund Period (2012)

The total monthly cost of NSP provision in May 2012 was $41,681 (Table 2). The cost per harm reduction contact was $44.72 while the cost per syringe distributed was $0.75. Nearly two-thirds (62%) of the total monthly costs were attributed to ancillary kit components (shown in Figure 2; $26,262) while syringes contributed 27% ($11,395) and 10% consisted of recurrent personnel, non-personnel, and capital costs. The cost of syringe distribution excluding ancillary kit components (personnel + other recurrent + capital + syringes only) during the GF period was $0.28 per syringe distributed.

For peer review only - http://bmjopen.bmj.com/site/about/guidelines.xhtml

For peer review only

Table 2: Capacity, optimum (May 2012) and average monthly costs of NSPs operating in Tijuana, Mexico (all costs in 2017 $USD)

	GF-period May 2012	Post-GF period 2015/2016 (average month)
**Unit of service**		
Harm reduction contacts per month	932	2,140
**Capital cost (monthly)**	**$778**	**$778**
Building/space*	$537	$537
Equipment	$241	$241
**Personnel‡ (monthly)**	**$2,503**	**$2,407**
Coordinators	$748	$748
Accountant	$94	$94
Counselor/Head of harm reduction services	$424	$424
Clinician (Nurse/Physician)	$698	$698
Health educators/Outreach workers	$539	$443
**Non-personnel recurrent costs (monthly)**	**$38,399**	**$4,947**
Syringes	$11,395	$1,853
Supplies	$178	$178
Utilities and other services‡	$564	$485
Ancillary harm reduction contents	$26,262	$2,431
**Total monthly cost§**	**$41,681**	**$8,131**
**Cost per harm reduction contact**	$44.72	$3.80
**Cost per syringe distributed including ancillary kit contents**	$0.75	$0.76
**Cost per syringe distributed excluding ancillary kit contents**	$0.28	$0.53

‡only includes the amount dedicated to providing harm reduction services
§may not sum to total due to rounding

NSP provision and utilization post Global Fund (2015/16)

Operations and provision of harm reduction services differed substantially during and after the GF withdrew support in 2015/16 (Figure 1B and Table 1). As shown in Figure 1B, geographic coverage of providing harm reduction services was sharply reduced after the GF withdrawal and limited mostly to the Zona Norte. Outreach personnel was reduced from 8 workers in 2012 to 4 workers in 2015/2016. Additionally, post-GF, the harm reduction kit contents were substantially reduced compared to the GF period (Table 1 and Figure 2). In 2015, the harm reduction kit contents included five syringes (three low-dead space and two high-dead space, compared to 60 low dead space syringes in 2012), three 3-ml vials of sterile water, five alcohol swabs, and three condoms. Additionally, post-GF, service provision was reduced by one day to five days per week. Despite

For peer review only - http://bmjopen.bmj.com/site/about/guidelines.xhtml

decreased geographic coverage and reduced opening hours, the number of kits distributed per month was higher in 2015 than in 2012. The NSP reported a mean of 2,140 monthly contacts in 2015/16 (3,170 contacts during highest volume month of 2015/16), compared to 932 in May 2012. However, because of the substantial decrease in syringes per kit, the total number of syringes distributed was substantially lower in 2015/16- an average of 10,700 syringes distributed per month in 2015/16. During the highest volume month in 2015/16, there were 15,850 syringes distributed compared to 55,920 in May 2012.

Cost of NSP provision post Global Fund (2015/2016)

The total monthly cost of the NSP was over 5-fold higher during 2012 compared to the average monthly cost during 2015/16 ($41,681 vs. $8,131 respectively), primarily due to the higher cost of the harm reduction kit during the GF period ($44.72 vs. $3.80 per kit, respectively). By comparison, the total monthly cost during the month of maximum provision, post-GF, was $10,193. The cost of just syringe distribution (excluding ancillary kit components) doubled from $0.28/syringe distributed in 2012 to $0.53/syringe distributed in 2015/16, mostly due to the reduction in syringes distributed and thus the higher personnel cost per syringe (Figure 3). However, after including ancillary kit components, there was no change in the cost per syringe distributed in 2015/16 ($0.76) compared to May 2012 ($0.75), because of the reduction in ancillary kit component expenditure. Similarly, there was little change in cost per syringe ($0.64) distributed when comparing to the maximum volume month in 2015/2016. Harm reduction kit (syringes + ancillary components) comprised 90% of the costs per syringe distributed during the GF period, whereas these items comprised only 51% in 2015/2016 (Figure 3).

Temporal trends in NSP access among PWID

Based on data from PWID in the El Cuete IV study, we calculated the mean predicted probabilities of accessing a NSP over the 17 three-month periods, which are shown in Figure S1 (Additional file 1). Overall, there was a significant increasing trend in the probability of accessing the NSP during the GF period, which peaked in September 2013 (51%, 95% CI: 42% - 59%). During the GF period, the mean log odds of accessing a NSP

For peer review only - http://bmjopen.bmj.com/site/about/guidelines.xhtml

increased by a factor of 0.17 (p-value < 0.001). The immediate change that occurred between the end of the last three-months of the GF period and the end of the first three months of the post-GF period was associated with a 0.73 reduction in the mean log odds of accessing a NSP in the past 6 months (p=0.02). During the post-GF period, the mean log odds of accessing a NSP decreased by a factor of 0.22 (p=0.002).

DISCUSSION

Our analysis is among the first to describe the cost of providing needle and syringe services in a Latin American setting and the first to specifically compare coverage and costs of needle and syringe during versus after withdrawal of GF support. We found dramatic declines in geographical coverage and number of syringes and ancillary kit components distributed post-GF withdrawal among one NSP provider in Tijuana, with concomitant declines in reported syringe access among PWID. Excluding ancillary kit components, cost per syringe distributed doubled post-GF; total cost per syringe (including kit components) remained similar across periods as ancillary components were dramatically reduced to cut costs. We expect to use both GF ("ideal") and post-GF ("current") NSP provision costs to inform future cost-effectiveness analyses of NSPs on reducing HIV incidence in LMIC.

Despite dramatic declines in volume, quality, and geographical coverage of NSP post-GF withdrawal, it was encouraging that NSP provision in both periods covered an array of services recommended by the WHO [12]. Among these, the WHO recommends multiple delivery modalities (NSPs operated both fixed sites and mobile outreach), and referral for first aid, drug treatment, voluntary HIV testing and treatment, diagnosis and treatment of STIs. While several of these services are available to NSP clients, evidence based drug treatment programs, such as opioid substitution therapy, have not been scaled up sufficiently in Tijuana.[17] We note that the WHO recommends that harm reduction kits include needles and syringes, condoms, filters, sterile water, swabs, spoons, puncture-proof containers, acidifiers, tourniquets, bleach and other disinfectants, and education material. Many of these items were provided during the GF era, and although this provision was drastically

For peer review only - http://bmjopen.bmj.com/site/about/guidelines.xhtml

reduced post-GF withdrawal, the kits still contained sterile water, alcohol swabs, and condoms. Future provision should emphasize increasing coverage of both needles and syringes as well as ancillary kit components for maximum prevention benefit.

While the longitudinal PWID cohort provided some external validation of our findings on diminished NSP provision after GF withdrawal, some caution is warranted when attempting to triangulate these results. Participants in the study did not specify from which NSP (nor from which geographical site) they received syringes, thus we cannot conclude with any certainty that the participants received their syringes from the NSP that we analysed during and post-GF. Despite this, we still found a highly significant reduced log odds of NSP utilization in the post-GF period. Overall, this finding is consistent with national level data from CENSIDA, which reported an 80% decrease in the number of syringes distributed per PWID from 2013 (19.7 syringes per PWID) to 2014 (3.9 syringes per PWID; see Figure S2).[18]

To our knowledge, our study is the first to estimate the cost of NSP provision in Latin America. Our reported cost per syringe distributed in Tijuana after the GF withdrew support ($0.76 per syringe distributed) is less than estimates in other high-income countries, but higher than other low-middle income country (LMIC) settings. For example, an analysis in the U.S. and Canada estimated a cost of $2.28 per syringe distributed, after inflating to 2017 USD.[19] In the U.K., cost per syringe/needle distributed ranged from $0.28 - $2.17[20] with similar cost per syringe distributed in Australia ($1.94).[21] These differences may be attributed to higher personnel costs which represented 66% of the costs in U.S. and Canadian NSPs, while this comprised approximately 30-40% in Tijuana in the post GF period. Conversely, the cost per harm reduction contact in Mexico was higher than in other LMICs, including Bangladesh ($0.42 in 2017 USD)[7] , China ($0.13 in 2017 USD)[22] and Russia ($0.38 in 2017 USD).[23] Despite a similar number of syringes distributed per month personnel costs were approximately ten times lower in China compared to Mexico in the post GF period. However, we cannot determine whether this was due to greater efficiency or fewer personnel.

For peer review only - http://bmjopen.bmj.com/site/about/guidelines.xhtml

Limitations

Our study has several limitations. First, we did not collect any client cost data so we cannot provide any non-service delivery costs, such as PWID transportation to the NSP site. This distinction may be relevant when comparing 2012 to 2015 since PWID were provided more syringes per harm reduction kit in 2012 and therefore may have not needed to access the NSP as frequently (we saw a correspondingly lower number of contacts per month during this period compared to post-GF). In addition, the geographic reach post-GF was much more limited, potentially substantially increasing transport costs for PWID. We are also uncertain about the number of unique PWID who accessed the NSP during the GF period since we only obtained the number of contacts (kits distributed).

Secondly, we report on changes in NSP provision during and post-GF support from one NSP in Tijuana, however other providers (one additional NSP and pharmacies) also provide sterile syringes.[24] However, interviews with the other NSP provider indicate that the reduction in services is likely generalizable. It is legal to obtain syringes from pharmacies, however PWID often report discrimination and pharmacists' refusal to sell syringes.[25] It is possible that reductions in syringe provision seen within our NSP site due to withdrawal of GF support could have been countered by increase provision of sterile syringes from pharmacies; however, self-reported data from the longitudinal cohort of PWID do not indicate this occurred (data not shown). Unfortunately, the El Cuete IV survey only began collecting information on number of sterile syringes obtained from any source (NSP, pharmacy, etc.) during the last month of Global Fund funding (December 2013) and therefore we were unable to assess individual-level trends before and after. We recognize the importance of collecting these data because previous findings indicate that individual level coverage affects HIV[26] and HCV risk[2 27], and these estimates could provide additional insight on monitoring temporal trends in NSP access and provision. Surveillance of clean syringe provision is critical since NSPs are often supported by donors, which could abruptly withdraw funding.

For peer review only - http://bmjopen.bmj.com/site/about/guidelines.xhtml

CONCLUSIONS

In Tijuana, Mexico, abrupt withdrawal of GF support was associated with dramatic declines in coverage and availability of needle and syringe provision, an effective[28] and cost-effective HIV prevention intervention.[29] NSP provision during the GF period in Tijuana involved greater access to sterile syringes at a similar cost per syringe distributed while also providing abundant ancillary items that promoted safe injecting and sexual practices. The withdrawal of multilateral donors has undoubtedly left an impact on other LMIC and is not unique to Mexico. For example Romania, which lost NSP funding from the Global Fund after joining the EU, saw a precipitous rise in new HIV infections shortly thereafter.[30] In terms of policy implications, it is becoming increasingly important for donors and governments to understand the financial and programmatic implications of abrupt donor withdrawal, and to appropriately plan for transition strategies which ensure that these services are not compromised in terms of quality, coverage, and monetary value.

DECLARATIONS

Ethics approval and consent to participate

Study procedures were approved by the institutional review boards at University of California San Diego Human Research Protections Program and Universidad Autónoma de Baja California and all NSP personnel provided written informed consent. The study protocol for the El Cuete IV was approved by the University of California San Diego Human Research Protections Program and El Colegio de la Frontera Norte (Tijuana).

Consent for publication

Not applicable

Availability of data and material

Costing spreadsheets are available upon reasonable request. El Cuete IV data cannot be shared due to the need to ensure the confidentiality of participants.

For peer review only - http://bmjopen.bmj.com/site/about/guidelines.xhtml

Competing interests

NM has received unrestricted research grants from Gilead unrelated to this work, and honoraria from Gilead and Merck. All other authors declare no competing interests.

Funding

This study was funded by grants from the National Institute on Drug Abuse (R01DA037773, K01DA043421, T32DA023356, R01DA015612), Fogarty International Center (R25TW009343), and by the University of California, San Diego, Center for AIDS Research (CFAR), an NIH-funded program (P30AI036214), which is supported by the following NIH Institutes and Centers: NIAID, NCI, NIMH, NIDA, NICHD, NHLBI, NIA, NIGMS, and NIDDK.

Authors' contributions

JC, JB, SS and NM conceived and designed the analysis and contributed to the writing of the manuscript. JC and JB conducted the analyses with input from JK, DA, TG, and PV. JB and PM collected cost data with assistance from RP, LAS, GR, and CM. All authors critically reviewed and approved the final version.

Acknowledgements

We thank the staff from the NSP and all respondents from the El Cuete IV study for participating.

For peer review only

For peer review only - http://bmjopen.bmj.com/site/about/guidelines.xhtml

REFERENCES

1. Aspinall EJ, Nambiar D, Goldberg DJ, et al. Are needle and syringe programmes associated with a reduction in HIV transmission among people who inject drugs: a systematic review and meta-analysis. *International journal of epidemiology* 2014;43(1):235-48. doi: 10.1093/ije/dyt243
2. Platt L, Minozzi S, Reed J, et al. Needle syringe programmes and opioid substitution therapy for preventing hepatitis C transmission in people who inject drugs. *The Cochrane database of systematic reviews* 2017;9:Cd012021. doi: 10.1002/14651858.CD012021.pub2 [published Online First: 2017/09/19]
3. Harm Reduction International. The Global State of Harm Reduction 2016, 2016.
4. Mathers BM, Degenhardt L, Phillips B, et al. Global epidemiology of injecting drug use and HIV among people who inject drugs: a systematic review. *The Lancet* 2008;372(9651):1733-45.
5. Kumaranayake L, Vickerman P, Walker D, et al. The cost-effectiveness of HIV preventive measures among injecting drug users in Svetlogorsk, Belarus. *Addiction* 2004;99(12):1565-76. doi: 10.1111/j.1360-0443.2004.00899.x
6. Vickerman P, Kumaranayake L, Balakireva O, et al. The cost-effectiveness of expanding harm reduction activities for injecting drug users in Odessa, Ukraine. *Sexually transmitted diseases* 2006;33(10 Suppl):S89-102. doi: 10.1097/01.olq.0000221335.80508.fa
7. Guinness L, Vickerman P, Quayyum Z, et al. The cost-effectiveness of consistent and early intervention of harm reduction for injecting drug users in Bangladesh. *Addiction* 2010;105(2):319-28. doi: 10.1111/j.1360-0443.2009.02755.x
8. Zhang L, Yap L, Xun Z, et al. Needle and syringe programs in Yunnan, China yield health and financial return. *BMC public health* 2011;11:250. doi: 10.1186/1471-2458-11-250
9. Mehta SR, Wertheim JO, Brouwer KC, et al. HIV transmission networks in the San Diego–Tijuana border region. *EBioMedicine* 2015;2(10):1456-63.
10. White EF, Garfein RS, Brouwer KC, et al. Prevalence of hepatitis C virus and HIV infection among injection drug users in two Mexican cities bordering the US. *Salud Publica Mexico* 2007;49(3):165-72. doi: Doi 10.1590/S0036-36342007000300001
11. Robertson AM, Garfein RS, Wagner KD, et al. Evaluating the impact of Mexico's drug policy reforms on people who inject drugs in Tijuana, B.C., Mexico, and San Diego, CA, United States: a binational mixed methods research agenda. *Harm Reduct J* 2014;11:4. doi: 10.1186/1477-7517-11-4
12. World Health Organization. Guide to starting and managing needle and syringe programmes. 2007
13. WHO and UNODC. Technical Guide for countries to set targets for universal access to HIV prevention, treatment and care for injecting drug users. 2009
14. Cunnama L, Sinanovic E, Ramma L, et al. Using Top-down and Bottom-up Costing Approaches in LMICs: The Case for Using Both to Assess the Incremental Costs of New Technologies at Scale. *Health Economics* 2016;25:53-66. doi: 10.1002/hec.3295
15. Dibble S. Gas price protest leads to shortages at Tijuana stations San Diego Union Tribune2017 [Available from: http://www.sandiegouniontribune.com/news/border-baja-california/sd-me-gas-shortage-tjuana-20170106-story.html accessed 12 April 2017.
16. Wagner AK, Soumerai SB, Zhang F, et al. Segmented regression analysis of interrupted time series studies in medication use research. *Journal of clinical pharmacy and therapeutics* 2002;27(4):299-309.
17. Werb D, Wagner K, Beletsky L, et al. Police bribery and access to methadone maintenance therapy within the context of drug policy reform in Tijuana, Mexico. *Drug Alcohol Depen* 2015;148:221-25.
18. CENSIDA. National Report on the Progress of the HIV/AIDS Response (Informe Nacional de Avances en La Respuesta al VIH y el SIDA) 2015 [Available from: http://www.censida.salud.gob.mx/descargas/ungass/GARPR_Mx2015.pdf accessed 25 May 2018.
19. Lurie P, Reingold AL. The Public Health Impact of Needle Exchange Programs in the United States and Abroad: Summary, Conclusions, and Recommendations. 1993
20. Platt L, Sweeney S, Ward Z, et al. Assessing the impact and cost-effectiveness of needle and syringe provision and opioid substitution therapy on hepatitis C transmission among people who inject drugs in the UK: an analysis of pooled data sets and economic modelling. 2017

For peer review only - http://bmjopen.bmj.com/site/about/guidelines.xhtml

21. Wilson D, Kwon A, Anderson J, et al. Return on investment 2: Evaluating the cost-effectiveness of needle and syringe programs in Australia. *Sydney: National Centre in HIV Epidemiology and Clinical Research, The University of New South Wales* 2009
22. Masaki E. CJ, Zhu Q., Chen X., Chen Y., Lu M. Cost -Effectiveness of Harm Reduction Interventions in Guangxi Zhuang Autonomous Region, China. *World Bank* 2007
23. Marseille E, Dandona L, Saba J, et al. Assessing the efficiency of HIV prevention around the world: methods of the PANCEA project. *Health services research* 2004;39(6p2):1993-2012.
24. Pollini RA, Lozada R, Gallardo M, et al. Barriers to Pharmacy-Based Syringe Purchase Among Injection Drug Users in Tijuana, Mexico: A Mixed Methods Study. *Aids Behav* 2010;14(3):679-87. doi: 10.1007/s10461-010-9674-3
25. Davidson PJ, Lozada R, Rosen PC, et al. Negotiating access: social barriers to purchasing syringes at pharmacies in Tijuana, Mexico. *Int J Drug Policy* 2012;23(4):286-94. doi: 10.1016/j.drugpo.2012.05.001
26. Bluthenthal RN, Anderson R, Flynn NM, et al. Higher syringe coverage is associated with lower odds of HIV risk and does not increase unsafe syringe disposal among syringe exchange program clients. *Drug Alcohol Depen* 2007;89(2-3):214-22.
27. Turner KM, Hutchinson S, Vickerman P, et al. The impact of needle and syringe provision and opiate substitution therapy on the incidence of hepatitis C virus in injecting drug users: pooling of UK evidence. *Addiction* 2011;106(11):1978-88.
28. Hurley SF, Jolley DJ, Kaldor JM. Effectiveness of needle-exchange programmes for prevention of HIV infection. *The Lancet* 1997;349(9068):1797-800.
29. Wilson DP, Donald B, Shattock AJ, et al. The cost-effectiveness of harm reduction. *Int J Drug Policy* 2015;26 Suppl 1:S5-11. doi: 10.1016/j.drugpo.2014.11.007
30. Csete J, Kamarulzaman A, Kazatchkine M, et al. Public health and international drug policy. *Lancet* 2016;387(10026):1427-80. doi: 10.1016/S0140-6736(16)00619-X [published Online First: 2016/03/30]
31. San Diego Department of Associated Governments. [Available from: https://www.sandag.org/index.asp?subclassid=100&fuseaction=home.subclasshome accessed 12 December 2018.
32. Instituto Metropolitano de Planeación: Ayuntamiento de Tijuana. [Available from: https://www.implantijuana.org/servicios/cartografia/carta-urbana-2010-2030/ accessed 12 December 2018.

For peer review only - http://bmjopen.bmj.com/site/about/guidelines.xhtml

FIGURES

Figure 1: Geographical coverage of needle and syringe program outreach sites (a) during GF period in May 2012 and (b) after GF withdrawal in 2015/16. GIS data of the major roads and administrative districts of Tijuana are publicly available from the San Diego Association of Governments (SANDAG)[31] and the Tijuana Metropolitan Institute of Planning, respectively.[32]

Figure 2: Harm reduction kit components per sterile syringe distributed during the Global Fund Period and after withdrawal of Global Fund. Note: Some items are only partially depicted since more syringes per item were distributed (e.g. during the GF period, three syringes were distributed per one condom).

Figure 3: Cost breakdown per syringe distributed at the NSP site (ancillary harm reduction components in brackets).

For peer review only

For peer review only - http://bmjopen.bmj.com/site/about/guidelines.xhtml

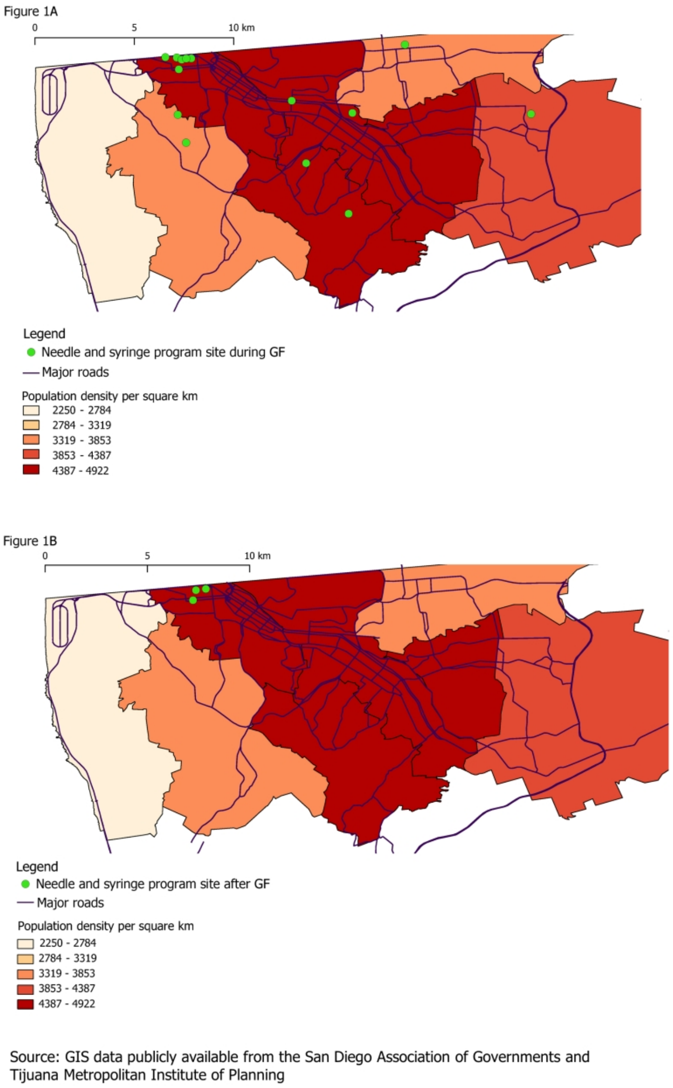

Figure 1: Geographical coverage of needle and syringe program outreach sites (a) during GF period in May 2012 and (b) after GF withdrawal in 2015/16. GIS data of the major roads and administrative districts of Tijuana are publicly available from the San Diego Association of Governments (SANDAG) and the Tijuana Metropolitan Institute of Planning, respectively.

190x338mm (300 x 300 DPI)

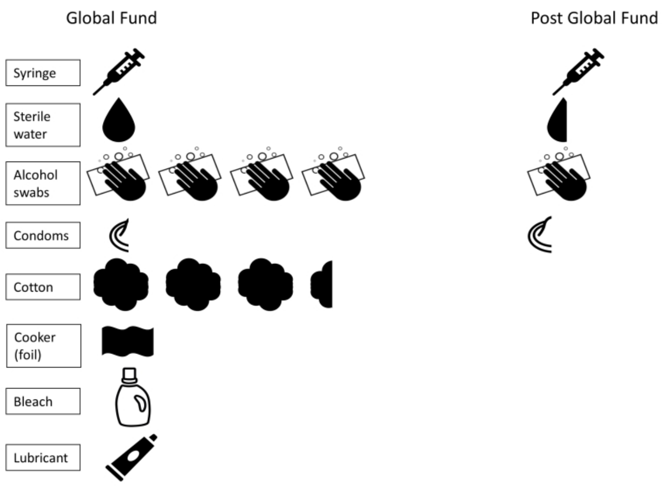

Figure 2: Harm reduction kit components per sterile syringe distributed during the Global Fund Period and after withdrawal of Global Fund. Note: Some items are only partially depicted since more syringes per item were distributed (e.g. during the GF period, three syringes were distributed per one condom).

338x190mm (300 x 300 DPI)

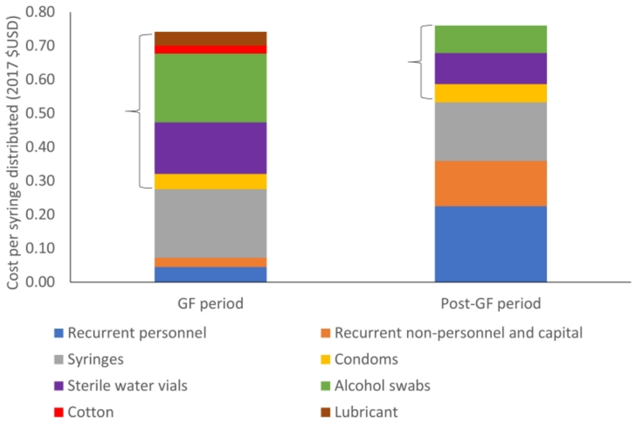

Figure 3: Cost breakdown per syringe distributed at the NSP site (ancillary harm reduction components in brackets).

338x190mm (300 x 300 DPI)

SUPPLEMENTAL APPENDIX

Additional details on the El Cuete cohort

Epidemiological data from the El Cuete IV prospective cohort study were used to inform our findings on needle and syringe program (NSP) utilization. Study details have been described previously.[1] Briefly, eligibility criteria at baseline included injecting drugs in the past month, being at least 18 years old, and not planning to move away from Tijuana over the next 30 months. Participants were recruited by street outreach from March 2011 – May 2013 (N=734), contributing 4,301 study visits in this analysis. Participants were followed biannually, however participants were not required to have injected drugs in the previous six months at their follow-up visits. All participants provided informed consent. The study protocol was approved by the University of California San Diego Human Research Protections Program and El Colegio de la Frontera Norte (Tijuana).

Measures

Our outcome of interest was accessing a NSP (either participants themselves or from someone else who obtained syringes from a NSP) in the past six months. The independent variable of interest was the calendar period, which we classified according to its respective GF period (during and post-GF). The surveys from all visits were sorted in ascending order based on the date of the interview. Next, they were grouped into three-month periods with each period corresponding to a different GF period (during and post-GF). We note that while the actual follow-up visits were spaced approximately 6 months apart and the surveys inquired about NSP utilization in the past 6 months, the calendar periods were grouped into three-month periods to allow us to capture and estimate any seasonal variation that might have occurred within each GF period. For simplicity, in terms of when El Cuete participants may have begun noticing the impact of the GF, we classified the GF period as beginning in March 2012 and ending before June 2014 given that participants were asked about behaviors in the past 6 months. While the

For peer review only - http://bmjopen.bmj.com/site/about/guidelines.xhtml

dates did not align exactly with the reported GF start and end times, we assumed that it most likely took the GF sponsored activities a few months to "ramp up" and "wind down". For example, prevention programs did not begin to be implemented in Tijuana until the third trimester of 2011.[2] In total, the data were divided into 17 evenly spaced three-month time periods, 9 of which occurred during the GF period and 8 in the post-GF period. On average, there were approximately 200-300 visits per three-month period.

Statistical analyses

We conducted segmented regression, a method used in the evaluation of intervention effects for interrupted time series data.[3] Logistic regression with fixed and random effects was used to estimate the mean log (odds of accessing a NSP) during the previous six months for each of the 17 three-month periods. Next, the mean log odds for each period estimated by the logistic regression model were used as the outcome variable in a segmented regression analysis, to predict the trend of accessing a NSP within each GF period as well as the level change from the GF period to the post-GF). Given the lack of independent error terms (as the errors of time series data are usually autocorrelated), a linear regression model was fitted to account for the autoregressive error. We tested for autocorrelation using the Breusch-Godfrey test and included first-order and second-order autoregressive terms to adjust for the effect of the positive autocorrelation.

RESULTS

The mean predicted probabilities of accessing a NSP over the 17 three-month periods are shown in Figure S1. Overall, there was a significant increasing trend in the probability of accessing the NSP during the GF period, which peaked in September 2013 (51%, 95% CI: 42% - 59%). During the GF period, the mean log odds of accessing a NSP in the increased by a factor of 0.17 (p-value < 0.001). The level change (the immediate change that occurred between the end of the last

For peer review only - http://bmjopen.bmj.com/site/about/guidelines.xhtml

three-months of the GF period and the end of the first three months of the post-GF period) from the GF period to the post-GF period was associated with a 0.73 reduction in the mean log odds of accessing a NSP in the past 6 months (p=0.02). During the post-GF period, the mean log odds of accessing a NSP decreased by a factor of 0.22 (p=0.002).

These trends were roughly consistent with national estimates of syringes acquired with GF support. In 2011, 534,573 syringes were distributed (92,070 financed by the GF [note: some sites may have been receiving funds prior to the NSP in Tijuana]), increasing to 1,904,961 syringes distributed (1,199,520 from the GF) in 2012. In 2013, 3,235,372 syringes were distributed with 78% provided by the GF (2,508,840). By 2014, the number of syringes distributed nationally declined to 643,320.[4]

For peer review only - http://bmjopen.bmj.com/site/about/guidelines.xhtml

Figure S1: Mean predicted probability of accessing a syringe exchange program in past six months among PWID in Tijuana. Error bars represent 95% confidence intervals. Shaded box represents the calendar period when the Global Fund operated in Mexico.

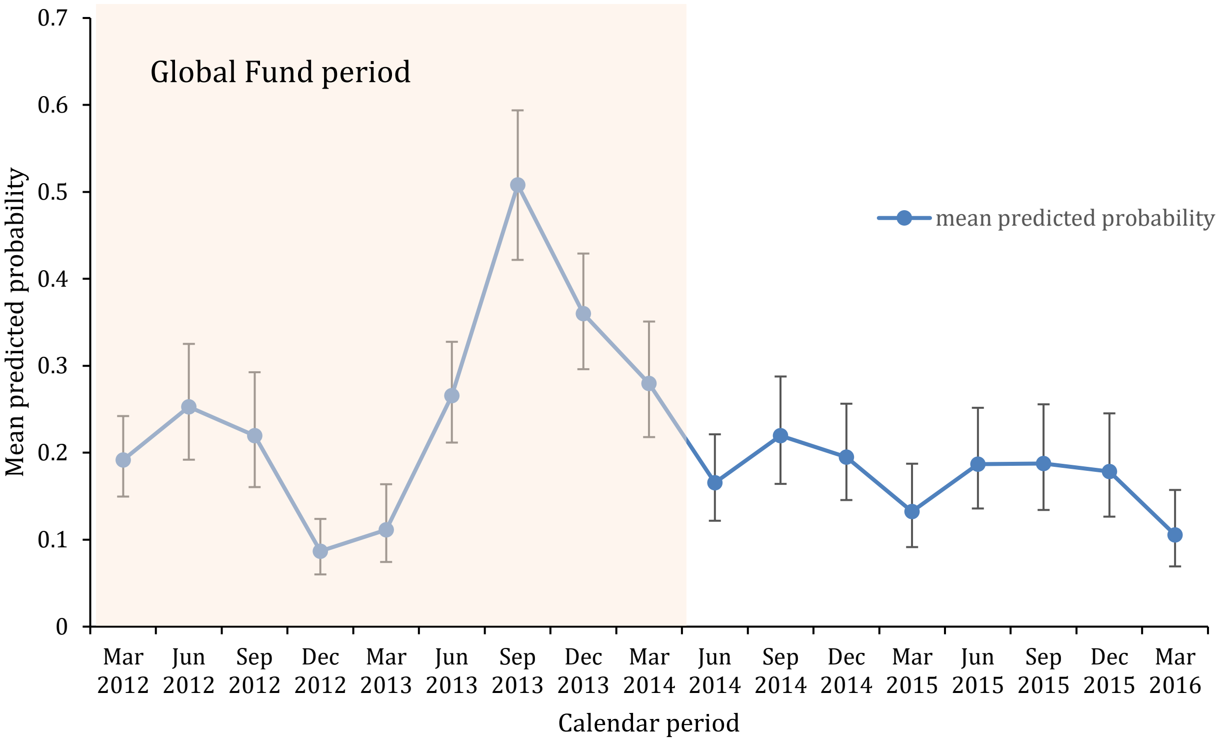

For peer review only - http://bmjopen.bmj.com/site/about/guidelines.xhtml

Figure S2: Number of syringes distributed in Mexico 2011 – 2014[4]

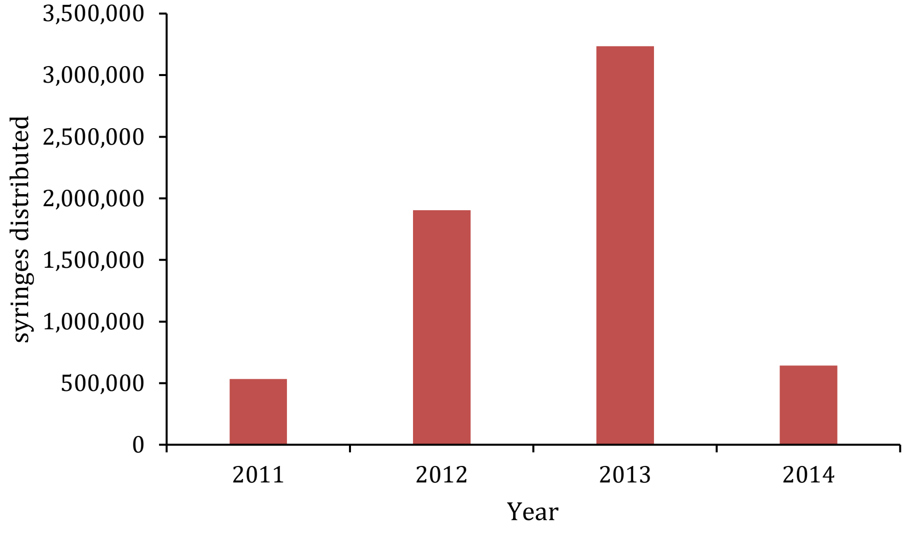

For peer review only

For peer review only - http://bmjopen.bmj.com/site/about/guidelines.xhtml

References

1. Robertson AM, Garfein RS, Wagner KD, et al. Evaluating the impact of Mexico's drug policy reforms on people who inject drugs in Tijuana, B.C., Mexico, and San Diego, CA, United States: a binational mixed methods research agenda. *Harm Reduct J.* 2014;11:4.
2. Fundación Mexicana para la Salud. "Fortalecimiento de las estrategias nacionales de prevención y reducción de daños dirigidos a HSH, HSH/TS, y UDI de ambos sexos". Plan de Implementación. 2011; http://funsalud.org.mx/vihfmfms/aprobados_fm/MX_Plan_Implentacion.pdf. Accessed 24 October, 2018.
3. Wagner AK, Soumerai SB, Zhang F, Ross-Degnan D. Segmented regression analysis of interrupted time series studies in medication use research. *Journal of clinical pharmacy and therapeutics.* 2002;27(4):299-309.
4. CENSIDA. National Report on the Progress of the HIV/AIDS Response (Informe Nacional de Avances en La Respuesta al VIH y el SIDA). 2015; http://www.censida.salud.gob.mx/descargas/ungass/GARPR_Mx2015.pdf. Accessed 25 May, 2018.

For peer review only
